# Combined effects of ultrasound and [bmim][cl] on heavy oil–solid separation: experiments and molecular dynamics simulations

**DOI:** 10.1016/j.ultsonch.2026.108019

**Published:** 2026-08-22

**Authors:** Liping Zeng, Xuanrui Wang, Ke Xu, Jinze Du, Yuxing Tan, Pindong Tan, Yu Sun, Jinjian Hou

**Affiliations:** a School of Chemistry and Chemical Engineering, Hainan University, Haikou 570228, China; b School of Chemical Engineering, Ocean and Life Sciences, Dalian University of Technology, Panjin 124221, China; c Zhejiang Institute of Tianjin University, Ningbo 315000, China; d School of Chemical Engineering, Tianjin University, Tianjin 300072, China

**Keywords:** Heavy oil, Ionic liquid, Ultrasound, Extraction, Molecular dynamics simulation

## Abstract

Efficient recovery of heavy oil from oil-bearing sands remains constrained by strong oil–solid adhesion, unfavorable interfacial conditions, and slow mass transfer in conventional solvent extraction. In this work, a solvent–ionic liquid–ultrasound intensified route was developed using [Bmim][Cl] as the key additive and toluene/p-xylene/m-xylene/o-xylene as representative solvents, with particular attention to the solvent-dependent interaction between ultrasound and [Bmim][Cl]. In the toluene system, the heavy-oil recovery increased from 90.6 wt% for single-solvent extraction to 92.5 wt% after the addition of [Bmim][Cl], corresponding to an increase of 1.9 wt%. The further introduction of ultrasound increased the recovery to 95.2 wt%, representing an additional increase of 2.7 wt% and an overall increase of 4.6 wt% relative to single-toluene extraction. Factorial interaction analysis demonstrated statistically significant synergy between ultrasound and [Bmim][Cl] in the toluene system (ΔR_syn_ = 2.10%, 95% CI: 0.38–3.82, p = 0.023) and a borderline statistically significant positive interaction in the p-xylene system (ΔR_syn_ = 2.00%, 95% CI: 0.01–3.99, p = 0.049), whereas the interaction estimates for the m-xylene and o-xylene systems were positive but not statistically significant (p = 0.198 and 0.158, respectively). The combined treatment also modified interfacial behavior: AFM measurements at the hydrophobized SiO_2_ model oil–solid interface showed that the interaction force decreased as the [Bmim][Cl] concentration increased, while interfacial tension decreased with increasing [Bmim][Cl] concentration under both sonicated and non-sonicated conditions. Because this model does not reproduce the mineralogical composition, surface morphology and roughness, or wetting heterogeneity of natural oil sands, the AFM force trend is used for controlled comparison and is not interpreted as a direct quantitative adhesion measurement on natural oil-sand surfaces. Wettability was improved in parallel, with the minimum water contact angle reduced to 24.7° under the combined treatment, consistent with a more water-wet post-treatment surface. Analysis of the properties and composition of the recovered oil showed that the highest C/H ratio of the recovered oil obtained under the combined toluene treatment was 9.08, which was slightly higher than that obtained in the single-toluene extraction system (8.73). Meanwhile, the SARA analysis showed that the resin and asphaltene mass fractions increased slightly from 27.96 and 21.93 wt% in the single-toluene extraction system to 28.56 and 22.39 wt% under the combined toluene treatment, respectively, with similar trends observed in the xylene systems. Given the small magnitude of these differences, this study uses the changes in the C/H ratio and SARA composition as auxiliary evidence for the trend of heavy-component release. Kinetic analysis indicated extraction acceleration, for instance, reducing the toluene equilibration time from approximately 20 min to approximately 10 min after [Bmim][Cl] addition. Molecular dynamics simulations further provide qualitative molecular-scale support for interpreting the experimental trends. In the simplified SARA representative molecular model, [Bmim][Cl] exhibits more pronounced local association with resins and asphaltenes; these short-range correlations do not by themselves establish specific interaction energies. The MD results are mainly used to compare the differences in local associations between different SARA representative molecules and [Bmim][Cl] and to serve as qualitative molecular-scale evidence for explaining the experimental trends, rather than as quantitative simulations of real asphaltene aggregation/disaggregation behavior. Within the simplified model, the polycyclic and polar features of the asphaltene-like molecule were associated with more pronounced local contact with [Bmim][Cl], suggesting a possible influence on the surrounding microenvironment. Overall, the results are consistent with ultrasound and [Bmim][Cl] playing complementary roles in reducing interfacial restrictions and accelerating mass transfer, realizing faster and more efficient heavy-oil release and providing a practical foundation for designing intensified, potentially lower-impact extraction processes.

## 1 Introduction

Energy plays an important role in industrial development [1], [2], [3], [4]. Heavy oil and oil sands are important components of unconventional oil resources, but their efficient development is still limited by factors such as high oil-phase viscosity, strong oil–solid adhesion, and slow interfacial mass transfer [5], [6], [7], [8]. Compared with conventional crude oil, heavy oil usually contains a high proportion of resins and asphaltenes [9], [10]. These components are rich in aromatic structures, polar groups, and heteroatomic sites, which are easily adsorbed on mineral surfaces through van der Waals forces, π–π interactions, polar interactions, and hydrogen bonding, thus forming a stable residual oil film [11], [12], [13]. Therefore, weakening heavy-oil adhesion to solid particles and promoting oil-phase migration from the solid surface to the solvent phase are key to improving heavy oil–solid separation.

At present, the separation technologies for heavy oil and oil sands mainly include hot alkaline washing, thermal treatment, solvent extraction, and reversible solvent extraction [14], [15], [16]. Hot alkaline washing can promote the detachment of asphaltenes from the surface of sand grains by changing the surface properties of oil sands, but this method usually has problems such as equipment corrosion, high wastewater treatment pressure, and limited adaptability to strongly hydrophobic oil sands. Thermal treatment methods can improve oil-phase fluidity by reducing the viscosity of heavy oil, but they are often accompanied by high energy consumption and may cause coke formation or structural transformation of heavy components [17], [18], [19], [20].

In addition to conventional heat treatment, thermally enhanced in-situ extraction technologies, such as steam huff and puff, steam flooding/steam drive, steam-assisted gravity drainage, and in-situ combustion, are important technologies for recovering heavy oils at the reservoir scale. Among them, in-situ combustion generates heat through low-temperature oxidation and high-temperature oxidation reactions in the reservoir, which can reduce the viscosity of heavy oil and promote a certain degree of in-situ modification. In recent years, research on metal complexes and catalyst-assisted in-situ combustion has further shown that catalysts can regulate the kinetics of heavy oil oxidation reactions and reduce the activation energy of high-temperature oxidation reactions [21], [22]. However, these thermal recovery methods typically require high-temperature conditions, air or oxygen injection, stable control of the combustion front, and reservoir adaptability assessment, and may cause compositional changes such as oxidation of heavy components, cracking, or coke formation. In contrast, the combined solvent–[Bmim][Cl]–ultrasound system proposed in this paper mainly targets the extraction and separation process of oil sands or oil-containing solids at low bulk-phase temperatures. Its goal is not to replace reservoir-scale thermal EOR, but to promote oil-film stripping, weaken oil–solid adhesion, and facilitate heavy-component release under milder conditions through ionic-liquid interfacial regulation and ultrasonic physical enhancement.

In heavy oil extraction and quality improvement research, thermochemical strengthening methods typically reduce oil-phase viscosity and improve fluidity by regulating oxidation reactions, cracking processes, and transformation of heavy components. Khelkhal et al. used micron- and nano-scale MnO catalysts to intensify in-situ combustion of heavy oil, finding that MnO/SiO_2_ composites can improve catalyst dispersion and significantly promote reaction rates in high-temperature oxidation zones, supporting combustion-front stability and improved thermal recovery efficiency [23]. Djouadi et al. compared the effects of Mn(acac)_3_ and manganese oil catalysts, showing that both catalysts can reduce reaction temperature and activation energy during the low-temperature and high-temperature oxidation stages of heavy oil, with manganese oil catalysts showing a more pronounced kinetic effect [24]. Khelkhal et al. further pointed out that catalysts derived from cobalt tall oil and cobalt sunflower oil can accelerate heavy oil oxidation and reduce the reaction energy barrier by providing active sites, promoting oxygen activation, and altering reaction pathways [25]. Djouadi et al. found that Fe(acac) _3_ can generate magnetite in situ under high-temperature conditions and promote Fe^2+^/Fe^3+^ electron transfer, thereby enhancing the high-temperature oxidation process of heavy oil [26]. Additionally, Galiakhmetova et al. used metallic sodium nanoparticles to assist hydrothermal treatment, promoting the conversion of kerogen and resin–asphaltene components, increasing the proportion of light saturated hydrocarbons, and reducing the content of heavy sulfur components [27]. The above studies indicate that thermal extraction and catalytic hydrothermal treatment mainly rely on high-temperature oxidation, cracking, or chemical conversion to improve the fluidity of heavy oil. In contrast, the combined aromatic solvent–[Bmim][Cl]–ultrasound system constructed in this paper mainly targets low-temperature extraction and separation of oil sands and oily solids. Through solvent dissolution, ionic-liquid interface regulation, and ultrasonic mass transfer intensification, it promotes oil film stripping and heavy oil migration. Its targets, temperature conditions, and strengthening mechanisms differ from reservoir-scale thermochemical recovery methods.

From this perspective, solvent extraction can achieve the dissolution and migration of heavy oil under relatively mild conditions and has good solubility for aromatic components, resins, and asphaltenes. However, single-solvent extraction is still limited by problems such as a slow solvent penetration rate, insufficient oil-film detachment, and difficulty in disrupting the aggregate structure of heavy components. Therefore, it is necessary to introduce field enhancement or functional additives to further improve the separation efficiency.

Ultrasound has been widely used to enhance multiphase mass transfer and petroleum-related separation processes through acoustic cavitation, acoustic streaming, microjets, and local shear [19], [28], [29]. For the process of heavy oil–solid separation, ultrasonic effects are expected to improve the extraction rate by thinning the solid–liquid interfacial diffusion boundary layer, promoting solvent penetration into oil-containing pores, disturbing the residual oil film, and enhancing the migration of dissolved oil into the bulk solvent phase. However, the effect of ultrasound itself is mainly reflected in the enhancement of physical mass transfer and mechanical stripping, and its ability to regulate the oil–solid interfacial energy barrier, mineral surface wettability, and resin/asphaltene aggregation structures is still limited. Therefore, it is difficult to fully explain the whole process of heavy-component release and interfacial adhesion weakening in complex heavy oil systems by relying on ultrasound alone.

Ionic liquids have been gradually used in petroleum-related separation processes in recent years due to their structural tunability, low volatility, surface activity, and strong solvation ability [30], [31], [32], [33], [34], [35]. Liaqat et al. [36] studied the application of hydrophobic ionic liquids in water extraction and oil removal, and found that by adjusting the structure and operating conditions of ionic liquids, particularly the cation structure and alkyl-chain length, the oil-phase removal efficiency can be significantly improved. Hu et al. [37] research indicated that ionic liquids and deep eutectic solvents could improve the heavy oil recovery, and there is a close relationship between the physicochemical properties of ionic liquids and oily sludge extraction efficiency. Mahtar et al. [38] synthesized lignin sulfonate-based ionic liquids and used them for asphaltene dispersion. The results showed that this type of ionic liquid could reduce the size of asphaltene particles and showed potential to regulate the aggregation behavior of heavy components. Zambom et al. [39] studied the role of the anionic structure of imidazolium ionic liquids in the processing of fuels and terpenes, and found that the anion composition can affect the separation performance by adjusting the polarity of the solvent. These studies show that ionic liquids have application potential in petroleum separation and heavy-component regulation, but most studies focus on oil–water separation, oily sludge extraction, or asphaltene dispersion, and the understanding of how ionic liquids affect the release process of different heavy oil components in the oil sands system is still insufficient.

As shown in Table 1, studies more directly related to heavy oil recovery and oil sands separation further show that ionic liquids can promote oil-phase release through wettability regulation, interfacial tension reduction, capillary force weakening, and oil–solid interaction weakening. Tafur et al. [40] evaluated the role of surface-active ionic liquids such as [C_12_mim][Br], [C_12_Py][Cl], and [C_16_Py][Cl] in smart water systems for low-temperature carbonate reservoirs, and found that they can cooperate with smart water to improve crude oil recovery. Sakthivel et al. [41] combined experiments and molecular simulations to study the enhanced oil recovery effect of [PF_6_]⁻- and [BF_4_]⁻-based ionic liquids in carbonate reservoirs, pointing out that ionic liquids can enter the oil–water interface and alter wettability, thus promoting oil-phase displacement. Rodríguez-Palmeiro et al. [42] studied the role of the phosphonium ionic liquid [P44414]Cl in surfactant-enhanced oil recovery, and found that it can reduce capillary forces and change the surface wettability of mineral solids. Hou et al. [43] put forward a new concept of ionic liquid-enhanced solvent extraction, suggesting that imidazolium ionic liquids can weaken oil–sand interactions and improve wettability, thus promoting bitumen debonding and oil-phase extraction. Tourvieille et al. [44] employed near-infrared hyperspectral imaging to investigate the kinetics of ionic-liquid-assisted bitumen extraction. Their results indicated that solvent selection was the principal factor governing bitumen recovery, whereas the type and viscosity of the ionic liquid affected extraction kinetics and fines capture. Li et al. [45] demonstrated that ionic liquids can facilitate the transfer of petroleum fractions from solid surfaces to organic solvents by lowering interfacial free energy and preferentially interacting with mineral surfaces. However, excessive mutual solubility between the solvent and ionic liquid may increase the entrainment of petroleum components in the ionic-liquid phase. The above research provides an important basis for ionic liquid-assisted heavy oil separation, but there are still differences in the mechanisms of action reported in different studies: one viewpoint emphasizes the occupation of interfacial adsorption sites by ionic liquids and their wettability-regulation effect, while another viewpoint suggests that ionic liquids can directly interact with heavy oil components.

**Table 1:** Ionic liquids used in enhanced-oil-recovery and oil–solid-separation studies.

References	Ionic liquid structure	Petroleum type	EOR	Proposed oil–solid-separation mechanism
[40]	[C_12_mim]Br [C_12_Py]Cl [C_16_Py]Cl	Low temperature carbonate reservoir crude oil	24.6%	In low-temperature carbonate systems, surface active cationic ionic liquids collaborate with Smart Water
[41]	[BF_4_] − ILs [PF_6_] − ILs	crude oil sample from one of the Saudi Arabian reservoirs.	39.3%	Ionic liquids can easily enter the interface and change wetting, promoting oil displacement and increasing recovery rate
[42]	[P_44414_]Cl	Sahara crude oil	Increase by 8%	Reduce capillary force and change the wettability of mineral solids
[43]	[Bmmim][PF_6_] [C_12_mim][Ac] [C_12_mim][BF_4_] [Emim][Ac]	heavy oil / bitumen	93.9%	Ionic liquids weaken oil–sand interactions and improve wettability, thereby promoting bitumen detachment and extraction.
[44]	[Bmmim][BF_4_] [Amim][DCA] [Emim][SCN] [EtNH_3_][NO_3_] [Amim][SCN]	Oil sand	90%	Solvent selection primarily controlled bitumen recovery; ionic-liquid viscosity affected extraction kinetics, while selected ionic liquids facilitated fines capture.
[45]	[Emim][BF_4_]	Simulated Crude Oil Distillates (SARA)	/	Ionic liquids can promote transfer of petroleum fractions from solid surfaces to organic solvents, lower interfacial free energy, and increase entrainment of petroleum fractions in the ionic-liquid phase.

Although studies have shown that ionic liquids can promote the release of heavy oil or bitumen, three issues remain unresolved. First, existing studies pay more attention to the total recovery rate or the properties of a single interface, and there is no systematic comparison of the release behavior of SARA components such as saturates, aromatics, resins, and asphaltenes. Second, it is still difficult to distinguish the relative contributions of solvent dissolution, ionic liquid interfacial regulation, and heavy-component dispersion. Third, whether a statistically significant positive interaction occurs between ultrasound-induced interfacial renewal and ionic liquid-induced reduction of the interfacial energy barrier across different solvent environments, and how this solvent-dependent interaction affects oil-film stripping, the migration of heavy components, and the reconstruction of molecular aggregate structures, still requires a clear mechanistic explanation.

Molecular simulation provides a molecular-scale perspective for understanding the above macroscopic phenomena. Vatti et al. [46] pointed out that with the increasing difficulty of heavy crude oil extraction and processing, the use of atomic- and molecular-scale simulation methods to understand the aggregation and inhibition mechanisms of asphaltenes is of great significance for screening sustainable solvents and regulating heavy oil systems. Wu et al. [47] studied the adsorption and distribution behavior of heavy oil SARA components on quartz surfaces through molecular dynamics simulations, which provided a molecular basis for understanding the different affinities of saturates, aromatics, resins, and asphaltenes to mineral surfaces. Li et al. [48] further investigated the liberation of asphaltenes from the muscovite surface, demonstrating that molecular simulation can be used to elucidate the interfacial factors governing asphaltene detachment. However, there is still a lack of systematic research on the interactions among [Bmim][Cl], aromatic solvents, and different SARA representative molecules, especially how these molecular interactions correspond to the macroscopic heavy oil release behavior under the ultrasound-assisted ionic liquid–solvent extraction framework.

Therefore, this work should be positioned as an additive-assisted oil–solid extraction strategy at low bulk temperatures, rather than directly replacing reservoir-scale thermal extraction. Compared with thermally driven in-situ mining methods, this method places greater emphasis on interface regulation, oil film stripping, and selective component release under mild conditions. Large-scale energy consumption and engineering applicability require further evaluation.

Based on the current status of the above research, the scientific contribution of this paper is mainly reflected in three aspects: first, ultrasound and ionic liquids are no longer discussed as two independent enhancement factors, but the solvent-dependent interaction between them in the solvent extraction process is evaluated through systematic control experiments and statistical analysis; second, the macroscopic recovery efficiency is linked to interfacial properties, recovered-oil SARA composition, and extraction kinetics, clarifying the complementary contributions of mass-transfer acceleration, the decrease in AFM-measured interaction force at the hydrophobized SiO_2_ model oil–solid interface, and the observed small changes in recovered-oil resin and asphaltene mass fractions; finally, the local associations between [Bmim][Cl] and different SARA representative molecules are further investigated through molecular dynamics simulations, providing qualitative molecular-scale information on local association trends between [Bmim][Cl] and SARA representative molecules, which helps interpret the experimentally observed trends in recovered-oil composition and interfacial behavior.

Based on this, this study constructs a solvent–[Bmim][Cl]–ultrasound combined heavy oil–solid separation system. With toluene, p-xylene, m-xylene, and o-xylene as representative aromatic solvents, and [Bmim][Cl] as a functional ionic-liquid additive, the system systematically examines the recovery efficiency, viscosity, C/H ratio, SARA composition, contact angle, AFM-measured interaction force at the hydrophobized SiO_2_ model oil–solid interface, zeta potential, interfacial tension, and extraction kinetics under different enhancement methods. At the same time, the interactions among [Bmim][Cl], aromatic solvents, and SARA representative molecules, together with their relative molecular mobility and local structural evolution, are compared through molecular dynamics simulations. The overall research route is shown in Fig. S1.

## 2 Materials and methods

### 2.1 Materials

The carbonate asphalt rocks (oil sands) were collected from the weathered zone of Buton Island in Indonesia. The water, oil, and solid contents of the sample were 0.12 wt%, 27.63 wt%, and 72.25 wt%, respectively. Toluene, p-xylene, m-xylene, o-xylene, 1-butyl-3-methylimidazolium chloride ([Bmim][Cl]), n-heptane, methanol, and trichloroethylene were all of analytical grade and were provided by Shanghai Aladdin Biochemical Technology Co., Ltd. (Shanghai, China).

### 2.2 Oil sands extraction process

#### 2.2.1 Solvent extraction process

The carbonate asphalt rock (oil-sand) samples were dried in an oven at 100 °C for 24 h to remove residual moisture. A 5 g portion (m_1_) of the dried sample was dispersed in 30 mL of organic solvent (toluene, p-xylene, m-xylene, or o-xylene) and magnetically stirred at 800 rpm and 30 °C in a constant-temperature water bath for 60 min. The mixture was then centrifuged at 7000 rpm for 5 min. The supernatant contained the heavy-oil/solvent phase, whereas the lower layer contained the solids. The solvent was removed from the supernatant by rotary evaporation. The recovered heavy oil was subsequently vacuum-dried at 80 °C for 6 h at 0.02 MPa to remove residual solvent and then weighed (m_2_). Heavy-oil recovery was calculated using Equation (1).

(1) $$\text{Heavy oil recovery =}\;\frac{m_{2}}{m_{1}\times Oilcontent}\times 100\%$$

#### 2.2.2 Ultrasound-enhanced solvent extraction process

The procedure followed Section 2.2.1, except that extraction was performed for 60 min in a custom-built low-frequency standing-wave ultrasonic reactor equipped with two HNT-8AE-2520 (5 × 4) piezoelectric transducers (Hainertec (Suzhou) Co., Ltd., Suzhou, China). The recovered heavy oil was weighed, and the recovery was calculated as described above.

#### 2.2.3 Ionic-liquid-enhanced solvent extraction process

A mixture comprising 5 g of the pre-dried oil-bearing solid, 30 mL of organic solvent, and 5 mL of [Bmim][Cl] was magnetically stirred at 800 rpm and 30 °C for 60 min. Following centrifugation, the mixture typically separated into three layers: an upper heavy-oil/solvent phase, an intermediate [Bmim][Cl] phase, and a lower solid layer. The remaining procedure followed Section 2.2.1, and the recovered heavy oil was quantified. The ionic-liquid phase was collected for potential recycling after successive filtration, washing, and drying.

#### 2.2.4 Ionic-liquid- and ultrasound-enhanced solvent extraction process

As was shown in Table S1, A 5 g portion of the pre-dried oily solid, 30 mL of organic solvent, and 5 mL of [Bmim][Cl] was placed in the ultrasonic reactor described above. The mixture was sonicated at 20 kHz and 60 W for 60 min, and the temperature was at 30 °C. After extraction, the heavy oil was recovered and the heavy oil recovery was calculated. Toluene was used to study the effects of sonication conditions. The frequency was maintained at 20 kHz, the nominal power was varied from 20 to 80 W, and the treatment time was varied from 15 to 30 min.

### 2.3 Characterization

#### 2.3.1 Sand properties

After extraction, the residual sand was obtained, and then it was washed, dried and then used for particle size and X-ray fluorescence analyses. For particle size measurement, approximately 2.0 g of the dried sample was dispersed in deionized water and analyzed using a Bettersize2000 laser particle size analyzer (Dandong Bettersize Instruments Ltd., Dandong, China) after background correction and ultrasonic degassing; each sample was measured in triplicate, and the mean value was reported. For XRF analysis, approximately 2.0 g of the cleaned sand was dried at 100 °C for 1 h, ground into a homogeneous powder using an agate mortar, and analyzed with an S4 PIONEER X-ray fluorescence spectrometer (Bruker AXS GmbH, Karlsruhe, Germany) to determine the relative contents of the major inorganic components.

#### 2.3.2 Recovered-oil properties

The recovered-oil properties included the heavy oil recovery, viscosity, C/H ratio and SARA (saturates, aromatics, resins, asphaltenes) content. The viscosity of the extracted heavy oil was measured using the Brookfield DV-III + rotational viscometer. The test temperature was maintained at 55.0 °C, using SC4-18 cylindrical rotors, with a fixed speed of 10 rpm, corresponding to a shear rate of 13.2 s⁻^1^. Before testing, samples were equilibrated at 55 °C for 10 min and pre-sheared at the same speed for 60 s. After the torque and viscosity readings stabilized, values were recorded continuously for 60 s, and their mean was reported as the steady-state apparent viscosity. All samples were measured in triplicate under the same temperature, rotor, shear rate, and shear history. The carbon (C) and hydrogen (H) contents of the recovered heavy oil were quantified using an elemental analyzer (Thermo Scientific FlashSmart, Germany), and then the C/H ratio was calculated. The detailed SARA content measured was as follows. First, m g of recovered heavy oil was weighed, and then it was treated with n-heptane, and the asphaltenes would precipitate, and then be separated. Then, the saturates, aromatics and resins would be centrifuged, concentrated. The n-heptane, toluene, toluene/methanol mixture and trichloroethylene were used to recover the saturates, aromatics, and resins. The mass fraction (wt%) of each component (M_n_) was calculated using **Equation** (2).

(2) $${\text{M}}_{\text{n}}=\frac{m_{n}}{m}\times 100\%$$

where M_n_ and m_n_ are the mass percent and mass of SARA.

#### 2.3.3 Oil-solid/water interfacial measurements

After extraction, centrifuge the mixture to remove the upper oil/solvent phase and intermediate ionic liquid phase, and collect the lower solid residue. In order to minimize the interference of residual oil, solvents, and [Bmim] [Cl], the solid was repeatedly dispersed in a compatible organic solvent and centrifuged until the supernatant was clear and there were no visible oil phase residues. Then wash the solid repeatedly with deionized water, dry to constant mass, cool to room temperature in a dryer, gently grind, sieve, and press into flat circular particles under consistent conditions. Store the particles in a dryer and measure the water contact angle at consistent time intervals using an SL200B goniometer (KINO, USA), because this washing and pellet-preparation procedure alters the original residue surface, the contact-angle results were used only for comparison among post-treatment samples.

The zeta potentials of heavy oil and sand residues obtained under the different extraction conditions were measured using a Zetasizer Nano ZS (Malvern Instruments Corp., England). For heavy-oil measurements, 1 g of bitumen was dispersed in 100 mL of water by sonication for 30 min; the pH was adjusted to 8.5 using 0.1 M NaOH or HCl, and the dispersion was shaken for 2 h before measurement. For solid measurements, 2 g of fine residue was dispersed in 100 mL of water and adjusted to pH 8.5 using the same procedure. Each measurement was repeated at least four times, and the mean value was reported. Deionized water was used as the electrolyte.

The effects of [Bmim][Cl] concentration and ultrasound on heavy-oil/water interfacial tension were measured by the pendant-drop method using an SL200B instrument (Kino, Winslow, AZ, USA) at 25 °C. Heavy oil recovered with or without ultrasound was diluted in toluene to 1 wt%, and 50 ppm asphaltenes were added. The oil phase was injected into an aqueous phase containing 0–1000 ppm [Bmim][Cl] through a U-shaped needle connected to a microsyringe. The Young–Laplace equation was fitted to the axisymmetric drop profile, and the reported value was obtained after the fitted interfacial tension became stable.

Atomic force microscopy (AFM; Dimension Icon XR, Bruker) was used to compare normalized interaction forces (F/R) at a simplified oil–solid interface. Hydrophobized SiO_2_ microspheres bearing a diluted heavy-oil film were used as the probe, with a measurement velocity of 2 μm·s⁻^1^. Measurements were performed after extraction with toluene, p-xylene, m-xylene, or o-xylene, using 600 ppm [Bmim][Cl] where applicable, with and without ultrasound; the detailed procedure followed Refs. [49], [50], [51], [52], [53]. This controlled interface was selected to compare the relative effects of the different treatments. It does not reproduce the mineralogical composition, surface morphology and roughness, or wetting heterogeneity of natural oil sands. The measured forces are therefore reported as normalized AFM interaction forces, F/R (mN·m⁻^1^), at the hydrophobized SiO_2_ model oil–solid interface, rather than as direct quantitative adhesion forces of natural oil-sand surfaces.

#### 2.3.4 Extraction kinetics

Aliquots of the upper phase were collected at defined time intervals and analyzed using a UV-3600i-Plus spectrophotometer. Heavy-oil concentrations were determined from solvent-specific calibration curves. To compare the heavy oil release rates under various enhancement conditions, pseudo-first-order and pseudo-second-order kinetic models were employed to empirically fit the time-dependent recovery profiles. The pseudo-first-order empirical model is represented using **Equation** (3):

(3) $${\text{R}}_{t}={\text{R}}_{e}\left(1-e^{-k_{1}t}\right)$$

where $R_{t}$ is the recovery efficiency of heavy oil at extraction time t, $R_{e}$ is the equilibrium recovery efficiency obtained by fitting, and $k_{1}$ is the pseudo-first-order apparent rate constant. The pseudo-second-order empirical model is represented using **Equation** (4):

(4) $${\text{R}}_{t}=\frac{k_{2}{\text{R}}_{e}^{2}t}{1+k_{2}{\text{R}}_{e}t}$$

where k_2_ is the pseudo-second-order apparent rate constant. The above models are used to describe the fitting trends of heavy oil recovery efficiency over time in different systems. The model fitting quality is compared by the coefficient of determination, R2, but this paper does not directly interpret the high fitting quality of pseudo-first-order or pseudo-second-order models as strict physical adsorption, chemical adsorption, or real mechanistic transformation. The relevant parameters are only used as a quantitative basis for comparing apparent rate differences among different extraction systems.

### 2.4 Molecular dynamics simulation

**Fig. 1:**
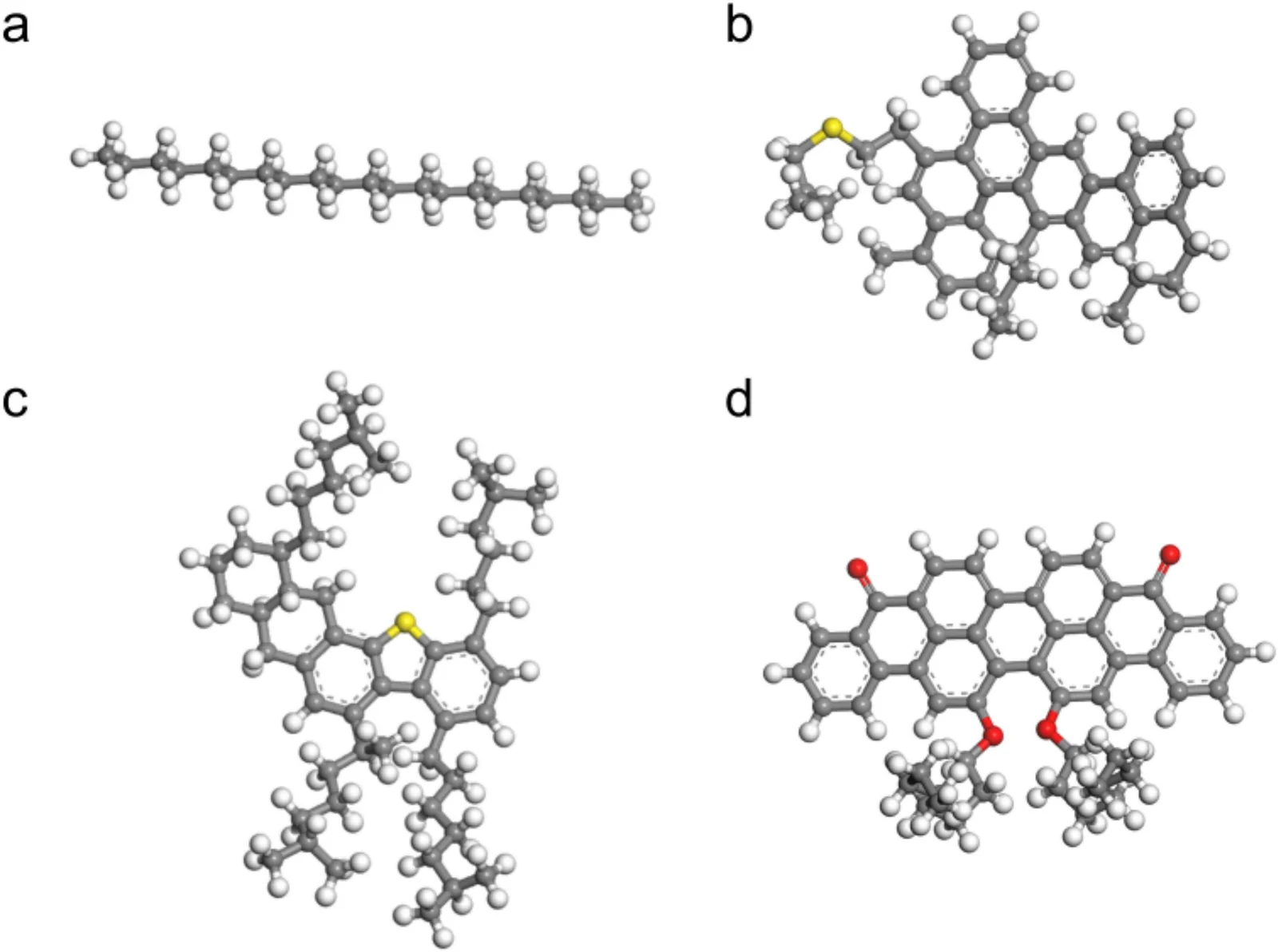
Molecular structures representing the heavy-oil SARA fractions [54]: (a) saturates (C_20_H_42_), (b) aromatics (C_46_H_50_S), (c) resins (C_50_H_80_S), and (d) asphaltenes (C_50_H_48_O_4_).

BIOVIA Materials Studio 2020 was used for molecular modeling and simulation of the different systems to investigate the interactions between [Bmim][Cl], solvents, and the SARA representative molecules. As shown in Fig. 1, the heavy oil was represented by four distinct molecule types based on its SARA fractions. The structures representing saturates, aromatics, resins, and asphaltenes were selected with reference [45], [48], [54], [55], [56]. A straight-chain alkane (C_20_H_42_) and a polyaromatic structure (C_46_H_50_S, MW 635 g·mol⁻^1^) were constructed to represent the saturates and aromatics, respectively. Additionally, a heteroatom-doped aromatic (C_50_H_80_S) and a bulky, polar approximately planar molecule (C_50_H_48_O_4_, MW 713 g·mol⁻^1^) were adopted for the resins and asphaltenes.

Periodic simulation boxes were generated using the Amorphous Cell module. To eliminate unphysical steric clashes and yield low-energy configurations, geometry optimization was conducted via the Forcite module with a maximum of 5,000 iterations. Subsequently, MD simulations were executed utilizing the COMPASS II force field and the Smart minimization algorithm.

Ball-and-stick molecular models of the SARA, solvents, and [Bmim][Cl] were constructed and individually subjected to energy minimization and structural optimization. The optimized molecular models were packed into an amorphous simulation cell using the COMPASS II force field. The numbers of molecules in each simulation system are listed in Table S2. Molecular dynamics simulations were performed using the Forcite module. The simulations were conducted in the NVT ensemble at 298 K using the COMPASS II force field and a Berendsen thermostat. A time step of 1 fs was employed, and the total simulation duration was 1000 ps. After the molecular dynamics simulations, the equilibrated simulation cell was analyzed by spatial layering. The convergence criteria were defined as an energy change of < 0.001 kcal·mol⁻^1^ and a maximum force of < 0.5 kcal·mol⁻^1^·Å⁻^1^. After the simulations, the Forcite analysis tools were used to calculate the mean square displacement (MSD), radial distribution functions (RDF), concentration distributions and other parameters. These descriptors were used only for comparison among systems prepared and simulated with the same protocol. Because the cells contained a limited number of representative molecules and the trajectories were 1000 ps long, the MSD results were not treated as statistically converged bulk-phase diffusion coefficients or as quantitative predictions of macroscopic transport.

## 3 Results and Discussion

### 3.1 Ultrasonic-power calibration

Calorimetry was used to quantify the acoustic power transferred by the 20 kHz standing-wave ultrasonic reactor. The calibration used the same reactor configuration and 35 mL liquid volume as the extraction experiments, with the external circulating thermostat turned off. The nominal generator power was set to 20, 40, 60, or 80 W, and the initial temperature increase was recorded continuously. Linear regression of the initial temperature–time region yielded the temperature-rise rate, from which the actual acoustic power was calculated using the system mass and specific heat capacity. As the nominal power increases from 20 W to 80 W, the actual sound power increases from 6.3 W to 25.7 W, and the corresponding sound power density increases from 0.180 W · mL^−1^ to 0.734 W · mL ⁻^1^. The sound energy conversion efficiency is 31.5% –32.3%, indicating a stable proportional relationship between the nominal generator power and the sound energy delivered to the liquid, but not equal.

The actual acoustic power was calculated using Equation (5):

(5) $$P_{\text{ac}}=mC_{p}{\left(\frac{\text{d}T}{\text{d}t}\right)}_{t=0}$$

The acoustic power density was calculated using Equation (6):

(6) $$P_{V}=\frac{P_{\text{ac}}}{V}$$

The energy input per unit volume was calculated using Equation (7):

(7) $$E_{V}=\frac{P_{\text{ac}}t}{V}$$

Table S3 reports the calorimetric acoustic power, power density, and energy input at the different nominal generator powers. Under the principal experimental condition of 60 W nominal power, the actual acoustic power was 19.4 ± 0.6 W and the acoustic power density was 0.554 ± 0.017 W·mL⁻^1^. Over the 20 min treatment, the acoustic energy input per unit volume was 665.1 ± 20.6 J·mL⁻^1^. These calorimetric values describe the dose delivered to the experimental system more directly than the generator panel setting alone. Because energy-transfer efficiency may depend on viscosity, density, heat capacity, ionic-liquid content, and reactor boundary conditions, the reported values apply to the calibrated liquid-loading conditions.

### 3.2 Sand properties

**Fig. 2:**
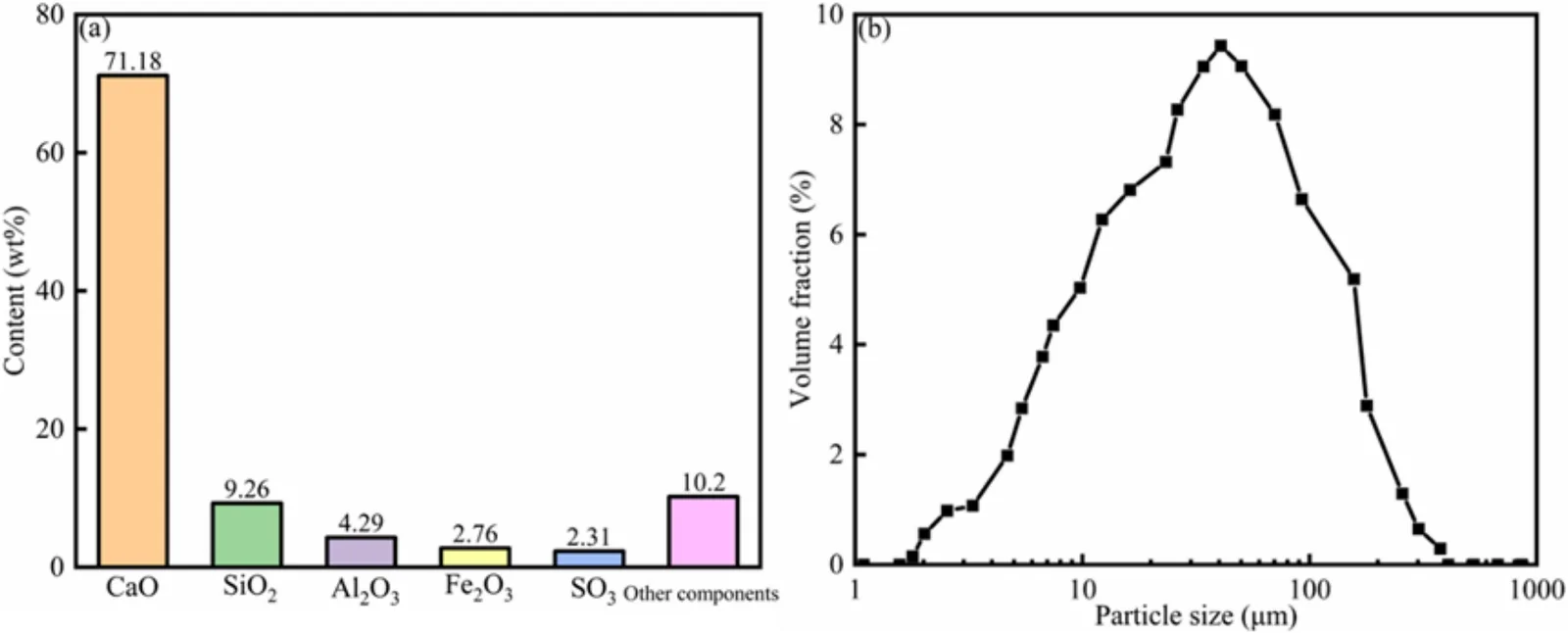
(a) Oxide composition (wt%) and (b) particle-size distribution of the sand residue after solvent extraction.

As shown in Fig. 2(a), the solid matrix of the Indonesian asphalt rock was calcium-rich, with CaO accounting for 71.18 wt% of the reported oxide composition. This advantage indicates that carbonate minerals are the main component of the sample. The content of Fe_2_O_3_ is 2.76% and 2.31%, respectively, while the total weight of other components is 10.20%. Due to the reporting of elemental oxide equivalents by XRF, it is necessary to confirm specific mineral phases through XRD. Fig. 2 (b) shows a wide particle size distribution, with most particles in the range of 5–200 μ m. The volume fraction increases with the increase of particle size, reaching a maximum of about 9.4% at 30–50 μ m, and then decreases; Above approximately 300 μ m, it approaches zero. Therefore, the sample is mainly composed of medium to fine particles. Their relatively large surface area and short internal diffusion distance may facilitate solvent penetration into particle voids and residual oil films. On the contrary, a higher content of fine particles may increase the oil solid contact area and retain heavy components through capillary effects, surface adsorption, and particle agglomeration.

In summary, the mineral composition and particle size distribution indicate that the behavior of the oil–water interface may be influenced by surface chemistry and particle scale structure. The polar sites on the surface of calcium rich minerals may interact with the oxygen-containing, sulfur-containing, and aromatic structures in resins and asphaltenes through acid-base, electrostatic, and hydrogen bonding interactions, while siliceous and aluminosilicate components increase surface non-uniformity. In addition, a broad particle-size distribution can produce complex pore structures and oil films at multiple length scales, potentially increasing mass-transfer resistance during conventional solvent extraction. These characterization results provide context for discussing possible changes in wettability, oil–solid interaction, solvent penetration, particle deagglomeration, and oil-film detachment; they do not by themselves establish the proposed mechanisms.

### 3.3 Recovered-oil properties

#### 3.3.1 Heavy oil recovery

**Fig. 3:**
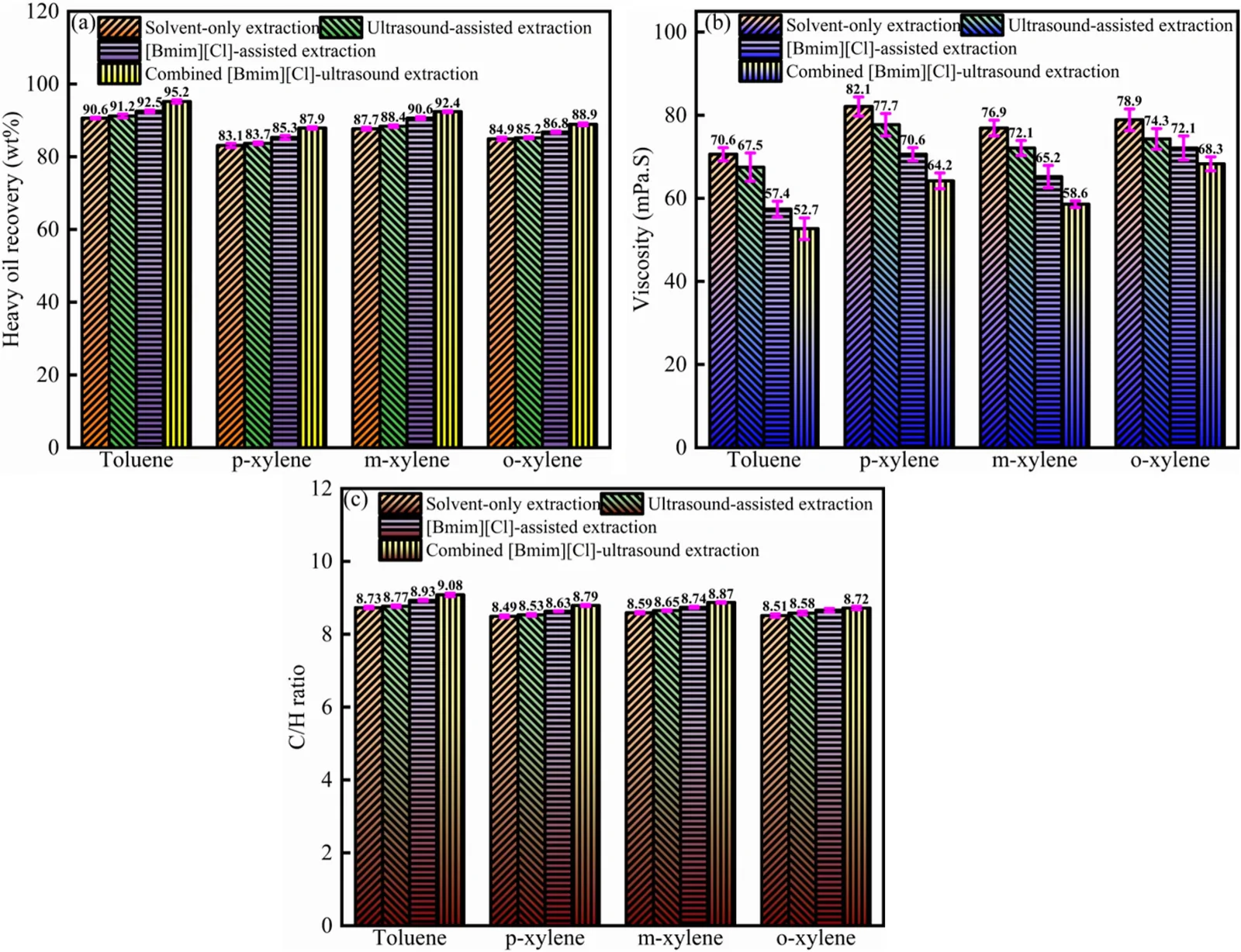
Recovered-oil properties: (a) recovery, (b) viscosity, and (c) C/H ratio.

Fig. 3**(a)** compares the heavy oil recovery under various enhancement methods. The recovery in the pure solvent systems ranges from 83.1 to 90.6 wt%. Although the independent addition of either ultrasound or [Bmim][Cl] improves the performance, the combined [Bmim][Cl]–ultrasound treatment consistently achieves the highest recovery across all tested solvents, reaching 95.2 wt% in the toluene system. Simultaneously, the combined treatment considerably decreases the viscosity of the recovered oil; the viscosities in the toluene, p-xylene, m-xylene, and o-xylene systems drop from 70.6, 82.1, 76.9, and 78.9 mPa·s to 52.7, 64.2, 58.6, and 68.3 mPa·s, respectively. This suggests that the incorporation of ultrasound and [Bmim][Cl] improves the fluidity and migration of the oil phase during extraction (Fig. 3**(b)**). Furthermore, as shown in Fig. 3**(c)**, the carbon-to-hydrogen (C/H) ratio of the heavy oil obtained from each system broadly follows a similar trend to the corresponding recovery. There was only a slight increase in the C/H ratio after the combined application of ultrasound and [Bmim][Cl].

**Fig. 4:**
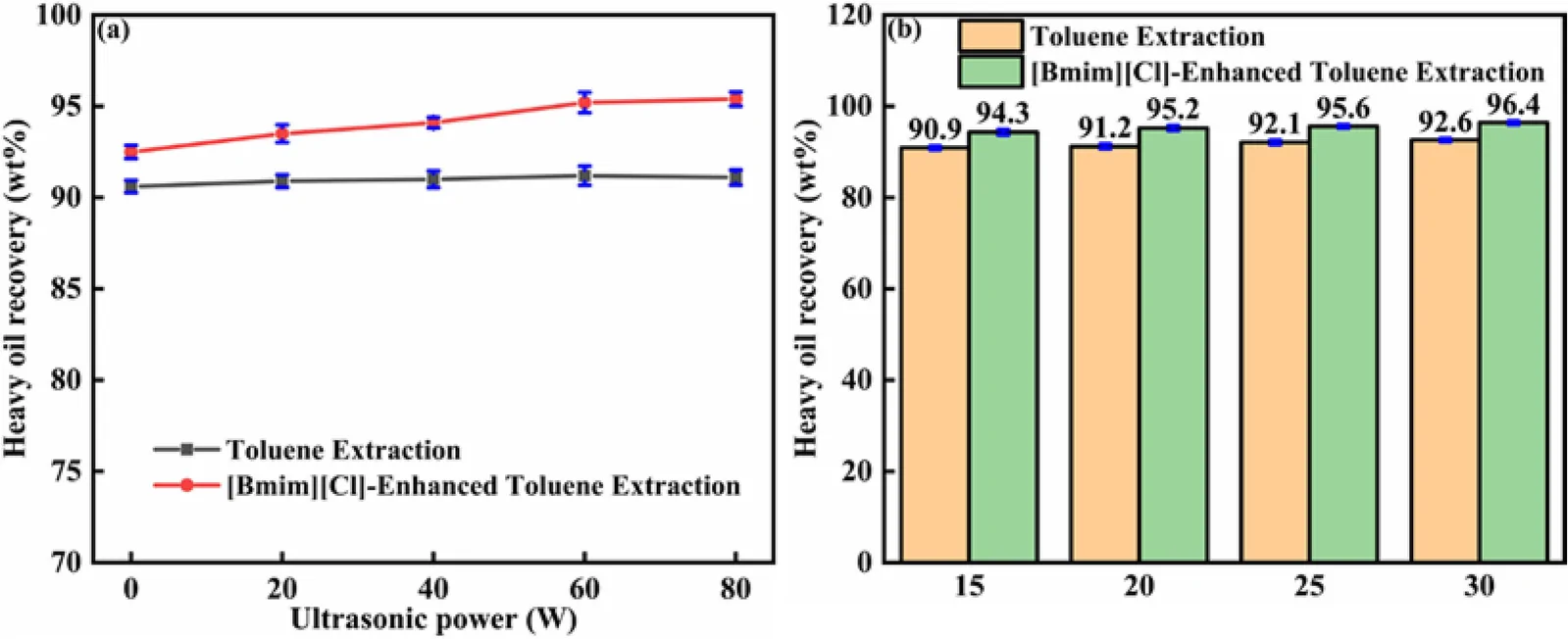
Effects of (a) nominal ultrasonic power and (b) ultrasound treatment time on heavy-oil recovery in the toluene systems.

Fig. 4 shows the effects of nominal ultrasonic power and treatment time on heavy-oil recovery in the toluene systems. As nominal power increased from 0 to 60 W, recovery in the [Bmim][Cl]-assisted system increased from approximately 92.5 to 95.2 wt% and then changed little above 60 W. The solvent-only toluene system remained near 91 wt% over the same power range. By increasing the ultrasonic treatment time, the recovery rate of toluene alone increased from 90.9 wt% to 92.6 wt%, and the recovery rate of the [Bmim] [Cl] auxiliary system increased from 94.3 wt% to 96.4 wt%. These numerical trends indicate that the response to ultrasound is different in the presence and absence of [Bmim] [Cl].

#### 3.3.2 SARA analysis

**Fig. 5:**
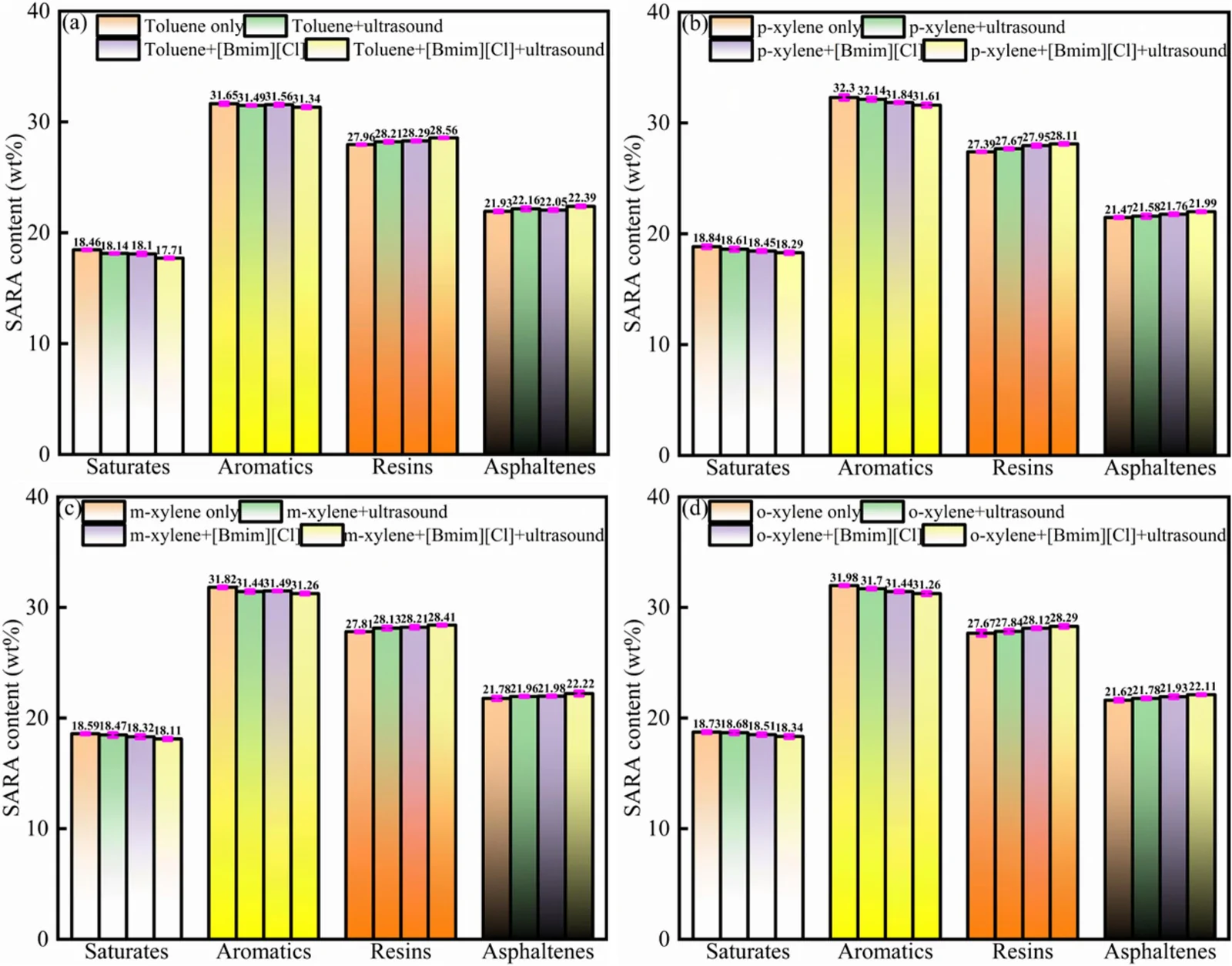
SARA mass fractions of recovered heavy oil in the (a) toluene, (b) p-xylene, (c) m-xylene, and (d) o-xylene systems.

Fig. 5 compares the SARA mass fractions of the heavy oil recovered under the different solvent and treatment conditions. Aromatics were the predominant fraction (approximately 31–32 wt%), followed by resins (approximately 27–29 wt%), asphaltenes (approximately 21–22 wt%), and saturates (approximately 18 wt%). Relative to solvent-only extraction, ultrasound or [Bmim][Cl] alone produced only small changes in the fractional distribution. Under the combined treatment, the saturates decreased slightly, whereas the resins and asphaltenes increased slightly.

#### 3.3.3 Statistical analysis

Ultrasound and [Bmim][Cl] were treated as two-level factors, and a 2 × 2 factorial design was constructed. Let R_0_ be the heavy oil recovery rate of the single solvent system, R_U_ be the recovery rate of the ultrasound-assisted solvent system, R_IL_ be the recovery rate of the [Bmim][Cl]-assisted solvent system, and R_U+IL_ be the recovery rate of the combined [Bmim][Cl]–ultrasound system.

The independent gains of ultrasound and [Bmim][Cl] are as follows:

(8) $$\Delta R_{\text{U}}=R_{\text{U}}-R_{0}$$

(9) $$\Delta R_{\text{IL}}=R_{\text{IL}}-R_{0}$$

If there is no interaction between the two factors, then the additive-prediction recovery is:

(10) $$R_{\text{add}}=R_{0}+\Delta R_{\text{U}}+\Delta R_{\text{IL}}=R_{\text{U}}+R_{\text{IL}}-R_{0}$$

The total gain of the combined system relative to a single solvent system is:

(11) $$\Delta R_{\text{abs}}=R_{\text{U + IL}}-R_{0}$$

The net interaction estimate of ultrasound and [Bmim][Cl] is calculated using the difference-in-differences form:

(12) $$\Delta R_{\text{syn}}=R_{\text{U + IL}}-R_{\text{U}}-R_{\text{IL}}+R_{0}$$

When $\Delta R_{\text{syn}}>0$, it indicates that the actual recovery rate of the combined system exceeds the additive prediction of the independent effects of the two factors, giving a positive interaction estimate; when $\Delta R_{\text{syn}}=0$, it indicates that the two mainly have an additive effect; when $\Delta R_{\text{syn}}<0$, it indicates that there may be antagonism between the two factors. A positive interaction estimate alone is not sufficient to establish synergy; statistical support requires a 95% confidence interval excluding zero and p < 0.05.

The descriptive proportion of the net interaction estimate relative to the total gain of the composite system is expressed as:

(13) $$C_{\text{syn}}=\frac{\Delta R_{\text{syn}}}{\Delta R_{\text{abs}}}\times 100\%$$

If each group contains n independent replicate measurements, the standard error of the interaction estimate is:

(14) $$SE_{\Delta R_{\text{syn}}}=\sqrt{\frac{s_{0}^{2}+s_{\text{U}}^{2}+s_{\text{IL}}^{2}+s_{\text{U + IL}}^{2}}{n}}$$

In the formula, s_0_^2^, s_U_^2^, s_IL_^2^, s_U+IL_^2^ are the standard deviations of recovery rates under four treatment conditions. The 95% confidence interval for the interaction estimate is calculated as:

(15) $$95\%CI=\Delta R_{\text{syn}}\pm {\text{t}}_{0.975,\nu }SE_{\Delta R_{\text{syn}}}$$

The interaction-test statistic is:

(16) $$t=\frac{\Delta R_{\text{syn}}}{SE_{\Delta R_{\text{syn}}}}$$

In the balanced 2 × 2 factorial design, this linear contrast has the same statistical significance as the ultrasound × [Bmim][Cl] interaction term in a two-way ANOVA. A two-sided test with p < 0.05 was used as the criterion for a statistically significant interaction.

As shown in Table 2, the combined [Bmim][Cl]–ultrasound treatment achieved the highest mean heavy-oil recovery in all solvents systems; however, the magnitude and statistical significance of the interaction varied with solvent identity. The solvent-only recoveries for toluene, p-xylene, m-xylene, and o-xylene were 90.6, 83.1, 87.7, and 84.9 wt%, respectively; after the combined treatment, they increased to 95.2, 87.9, 92.4, and 88.9 wt%, respectively. Compared to the corresponding single solvent system, the total absolute gains produced by the combined treatment were 4.6%, 4.8%, 4.7% and 4.0%, respectively. Thus, the combined treatment improved endpoint recovery across all solvents. Although the absolute gains of the xylene systems are slightly higher than the toluene gain, the toluene system, for which synergy was statistically supported, still achieved the highest final recovery rate, indicating that total gain amplitude and final recovery level belong to two different evaluation dimensions: the former is influenced by the base recovery rate, while the latter better reflects the solvent's overall dissolution and carrying capacity for heavy oil components.

**Table 2:** Independent effects, interaction estimates, and statistical tests of [Bmim][Cl] and ultrasound on heavy oil recovery rates in different aromatic solvent systems.

Statistical indicators	Toluene	p-xylene	m-xylene	o-xylene
Single solvent recovery R_0_ (wt%)	90.60 ± 0.72	83.10 ± 0.83	87.70 ± 0.76	84.90 ± 0.80
Ultrasound-assisted recovery R^U^ (wt%)	91.20 ± 0.65	83.70 ± 0.78	88.40 ± 0.72	85.70 ± 0.75
[Bmim][Cl]-assisted recovery R^IL^ (wt%)	92.50 ± 0.58	85.30 ± 0.69	90.60 ± 0.62	86.80 ± 0.68
Combined-treatment recovery R^U+IL^ (wt%)	95.20 ± 0.61	87.90 ± 0.66	92.40 ± 0.59	88.90 ± 0.64
Ultrasound-only gain ΔR^U^ (percentage points)	0.60	0.60	0.70	0.80
[Bmim][Cl]-only gain ΔR^IL^ (percentage points)	1.90	2.20	2.90	1.90
Additive prediction Radd (wt%)	93.10	85.90	91.30	87.60
Total combined-treatment gain ΔRabs (percentage points)	4.60	4.80	4.70	4.00
Net interaction estimate ΔRsyn (percentage points)	2.10	2.00	1.10	1.30
SE(ΔRsyn) (percentage points)	0.74	0.86	0.78	0.83
95% confidence interval (percentage points)	0.38–3.82	0.01–3.99	−0.71–2.91	−0.63–3.23
Interaction-test statistic, t	2.832	2.331	1.409	1.563
Interaction-test p value	0.023	0.049	0.198	0.158
Interaction estimate/total gain (%)	45.7	41.7	23.4	32.5
Statistical judgment	Statistically significant synergistic interaction	Borderline statistically significant positive interaction	Positive interaction estimate, not significant	Positive interaction estimate, not significant

From the perspective of single factor contribution, ultrasonic treatment increased the recovery rates of the four solvent systems by 0.6%, 0.6%, 0.7%, and 0.8%, respectively, while the addition of [Bmim][Cl] alone increased them by 1.9%, 2.2%, 2.9%, and 1.9%, respectively. Therefore, under the conditions of this study, the overall independent gain of [Bmim][Cl] was higher than that of ultrasound, indicating that under testing conditions, [Bmim][Cl] contributes more to endpoint recovery compared to using ultrasound alone. This difference is consistent with the role of [Bmim][Cl] in regulating wettability, interfacial forces, and local solvation environment. Although the independent gain of ultrasound is relatively small, its main function is not limited to improving the final balance recovery rate; It also includes diluting the diffusion boundary layer, promoting solvent permeation, accelerating oil film rupture, and continuously updating the oil liquid interface. Therefore, ultrasound act primarily as a kinetic strengthening factor, while [Bmim][Cl] simultaneously affects interfacial desorption resistance and the proportion of extractable heavy oil.

If the independent gain of ultrasound and [Bmim][Cl] is simply added, the theoretical sum recovery rates for toluene, p-xylene, m-xylene, and o-xylene systems are 93.1, 85.9, 91.3, and 87.6 wt%, respectively, all lower than the actual recovery rates of the corresponding combined systems. After further subtracting the independent contributions of the two factors, the net interaction estimates ΔR_syn_ for the four solvent systems were 2.1%, 2.0%, 1.1%, and 1.3%, respectively, all giving positive point estimates. However, a positive point estimate alone does not establish a statistically significant synergistic effect. The corresponding descriptive interaction-estimate proportions were 45.7%, 41.7%, 23.4%, and 32.5%, with the toluene system having the largest value. For the toluene system, this descriptive estimate was supported by a statistically significant interaction test and therefore represents a statistically supported synergistic gain.

From the perspective of statistical uncertainty, the net interaction estimate for the toluene system was 2.10%, with a 95% confidence interval of 0.38–3.82%, and the interaction effect test result was p = 0.023. Because the 95% confidence interval excluded zero and p < 0.05, the toluene system showed a statistically significant synergistic interaction under the tested conditions. These results suggest that ultrasound improve the accessibility of [Bmim][Cl] and toluene to viscous oil films and oil–solid contact regions, and accelerate the peeling and migration of unstable oil films through cavitation, microjets, and acoustic streaming, which may contribute to recovery beyond simple addition in the combined system.

The net interaction estimate of the p-xylene system was 2.00%, with a 95% confidence interval of 0.01–3.99%, and the interaction test gave p = 0.049. The lower bound of the confidence interval is close to 0, and the p value is close to 0.05, indicating a borderline statistically significant positive interaction, but the statistical evidence is weaker than that of the toluene system. Accordingly, this system is described as showing a borderline statistically significant positive interaction rather than a robust interaction effect. Its relatively wide confidence interval also indicates that under conditions of high base recovery rate and small absolute difference, fluctuations in parallel experiments have a significant impact on statistical conclusions.

The net interaction estimates of the m-xylene and o-xylene systems were 1.10% and 1.30%, respectively; the corresponding interaction-test p values were 0.198 and 0.158, respectively, and did not meet the significance standard of 0.05. Therefore, these two systems can only be identified as showing positive but statistically non-significant interaction trends at the mean level, because the observed differences may stem from random fluctuations in parallel trials. The improved recovery in the combined systems can be described primarily by the observed main effects of ultrasound and [Bmim][Cl], but existing statistical results are insufficient to prove that the two exhibit significant super-additive effects in m-xylene and o-xylene media.

Overall, the ΔR_syn_ values for all four solvents systems were positive, but the statistical strength of the interactions showed clear solvent dependence. The toluene system showed a statistically significant synergistic interaction, the p-xylene system showed a borderline statistically significant positive interaction, and the m-xylene and o-xylene systems showed only positive but statistically non-significant interaction trends. These results are consistent with [Bmim] [Cl] weakening of oil–water interface constraints, ultrasound enhanced interface updates, and interface mass transfer, and also indicate that relying solely on bar chart means or absolute gains cannot claim significant synergistic effects for all solvent systems. M−xylene and o-xylene were positive but statistically non-significant interaction trend.

### 3.4 Oil–solid/water interfacial analysis

**Fig. 6:**
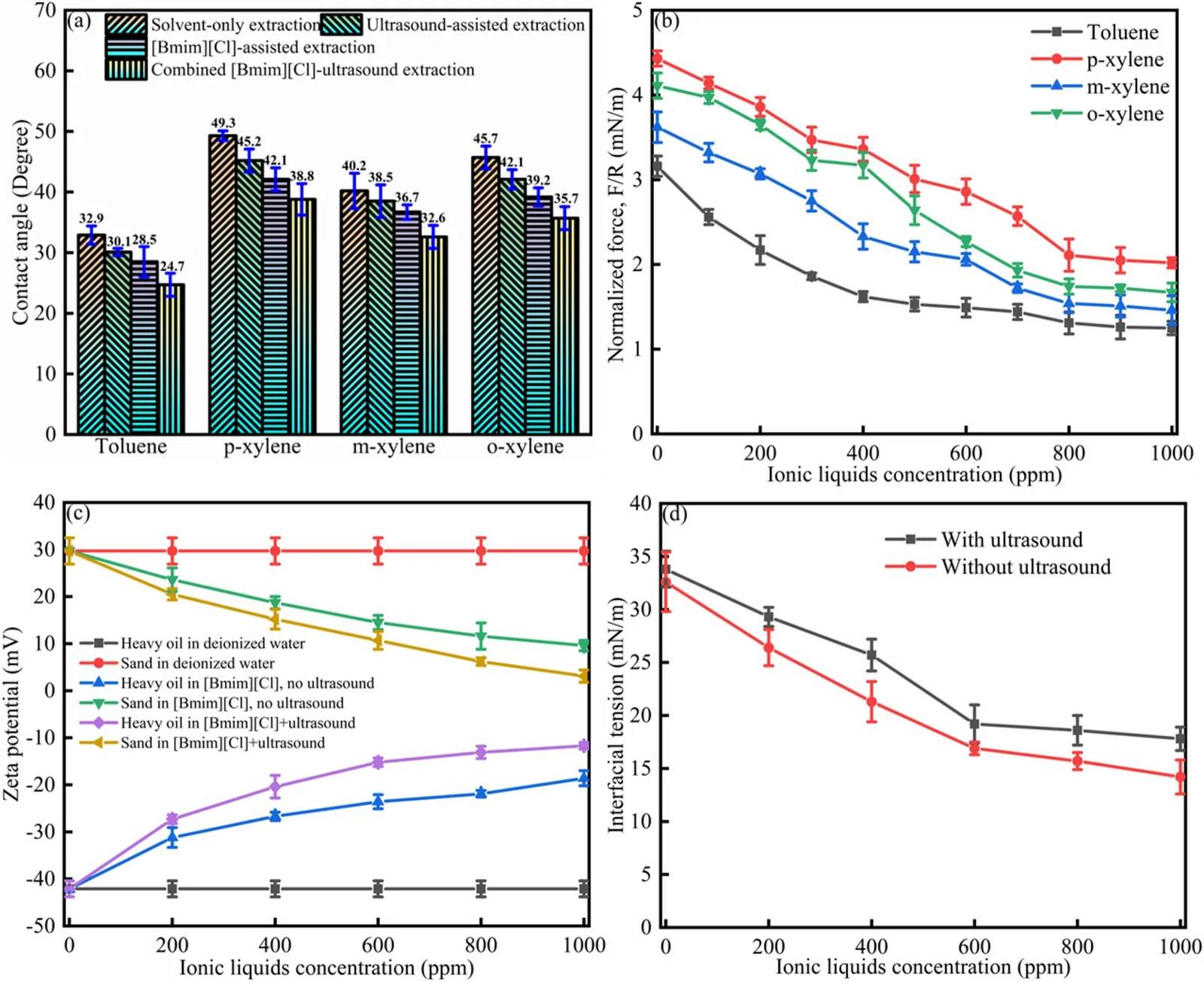
Oil–solid/water interfacial properties: (a) water contact angle; (b) normalized AFM interaction force, F/R, at the hydrophobized SiO_2_ model oil–solid interface under non-sonicated conditions; (c) zeta potential; and (d) interfacial tension under different treatments.

Fig. 6**(a)** compares the water contact angles of the solid residues obtained from different extraction systems. Relative to solvent extraction alone, the introduction of either ultrasound or [Bmim][Cl] reduced the contact angle. The combined [Bmim][Cl]–ultrasound system produced the lowest values for all four solvents, namely 24.7°, 38.8°, 32.6°, and 35.7° for toluene, p-xylene, m-xylene, and o-xylene, respectively. These results indicate that the combined treatment was associated with a more water-wet post-treatment surface. Across the different solvent systems, the contact angle generally followed the order p-xylene > o-xylene > m-xylene > toluene, suggesting that the extent of wettability improvement was dependent on the solvent used. As depicted in Fig. 6(b), the AFM-measured interaction force at the hydrophobized SiO_2_ model oil–solid interface decreased continuously with increasing [Bmim][Cl] concentration under non-sonicated conditions and approached a plateau at approximately 800 ppm. Fig. 6(c) shows the changes in the zeta potentials of heavy oil and sand surfaces under different conditions. In deionized water, the zeta potentials of heavy oil and sand remained at approximately − 42 and + 30 mV, respectively, indicating that the two surfaces carried opposite charges and were therefore likely subject to an electrostatic attractive tendency. As the [Bmim][Cl] concentration increased from 0 to 1000 ppm, the zeta potential of heavy oil became progressively less negative, whereas that of sand gradually decreased from positive values, with both surfaces shifting toward electrical neutrality. In the absence of ultrasound, the zeta potentials of heavy oil and sand in the 1000 ppm [Bmim][Cl] system reached approximately − 19 and + 9 mV, respectively. After ultrasound was introduced, the corresponding values further shifted to about − 12 and + 3 mV. Compared with [Bmim][Cl] alone, the additional shifts of both surface potentials toward neutrality were associated with the combined treatment; these data alone do not establish the molecular origin of the change. As illustrated in Fig. 6(d), the interfacial tension under both sonicated and non-sonicated conditions decreases continuously as the [Bmim][Cl] concentration increases from 0 to 1000 ppm. A pronounced decline is observed within the 0–600 ppm range, after which the trend gradually plateaus. At equivalent concentrations, the interfacial tension in the non-sonicated systems is generally marginally lower than that in their sonicated counterparts, suggesting that the [Bmim][Cl] concentration serves as the primary driver for interfacial tension reduction.

**Fig. 7:**
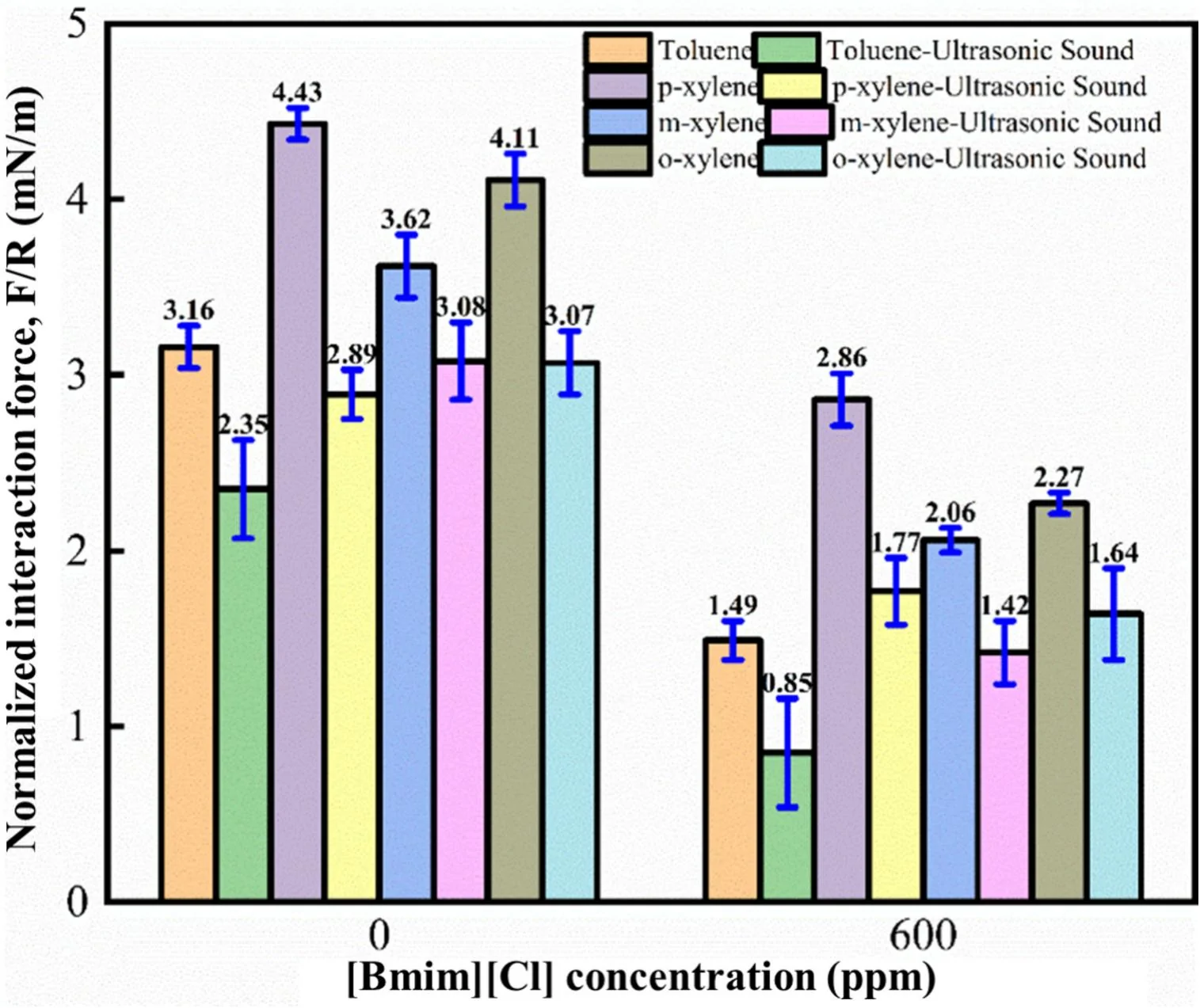
Effects of [Bmim][Cl] and ultrasound on the normalized AFM interaction force, F/R (mN·m⁻^1^), at the hydrophobized SiO_2_ model oil–solid interface.

According to Fig. 7, within the hydrophobized SiO_2_ model oil–solid interface, the addition of 600 ppm [Bmim][Cl] further decreased the normalized AFM interaction forces (F/R) to 1.49, 2.86, 2.06, and 2.27 mN·m⁻^1^. When [Bmim][Cl] was coupled with ultrasonication, the normalized model-interface forces decreased to 0.85, 1.77, 1.42, and 1.64 mN·m⁻^1^, respectively, representing reductions of approximately 73.1%, 60.0%, 60.8%, and 60.1% compared with their respective pure-solvent systems. These AFM results show that [Bmim][Cl] and ultrasonication lowered the measured interaction force within this model system, with the largest reduction observed for toluene. Because the probe surface is a simplified hydrophobically modified SiO_2_ surface, the force values are suitable for controlled comparison among the tested treatments but there still exists the differences between the simplified hydrophobically modified SiO_2_ surface and the natural oil sands minerals surface.

### 3.5 Extraction kinetics

#### 3.5.1 Pseudo-first- and pseudo-second-order models

**Fig. 8:**
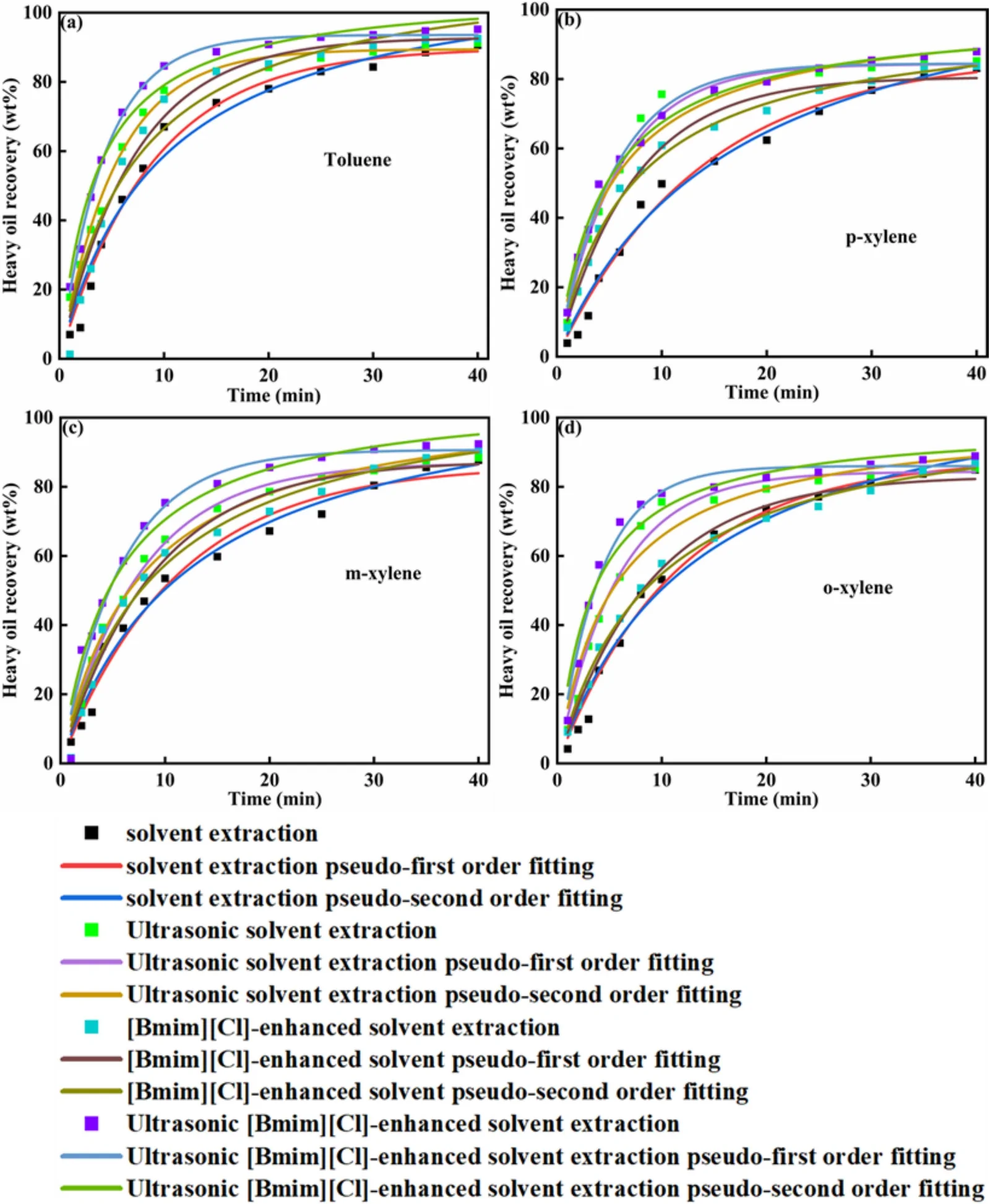
Time-dependent heavy-oil recovery and empirical kinetic fits for (a) toluene, (b) p-xylene, (c) m-xylene, and (d) o-xylene systems.

The time-dependent heavy oil recovery curve and empirical dynamic fitting are shown in Fig. 8. In all systems, the recovery rate initially increases rapidly, followed by smaller increments, and approaches the platform in approximately 30–40 min. This behavior is described empirically as the initial rapid mass transfer stage followed by a slower equilibrium. Compared to solvent extraction alone, [Bmim] [Cl] or ultrasound typically increases the apparent extraction rate and endpoint recovery rate, while combined treatment produces the largest initial increase and the highest plateau. These observations are consistent with faster oil film separation and oil phase transport, but empirical fitting alone cannot determine a unique rate control mechanism. Among the tested solvents, toluene had the highest overall recovery rate.

Table 3 summarizes the empirical kinetic parameters of the toluene system. After adding [Bmim] [Cl], ultrasound, or both, the apparent rate constant usually increases. In systems containing [Bmim] [Cl] and ultrasound, the pseudo first order model provides a higher R ^2^ value than the pseudo second order model, thus providing a closer description of the recovery curve. This numerical comparison does not establish a mechanical transition from second-order dynamics to first-order dynamics.

**Table 3:** Empirical kinetic fitting parameters for heavy-oil recovery in toluene-based extraction systems.

Models	Parameters	Toluene	Toluene + [Bmim][Cl]	Toluene + ultrasound	Toluene + [Bmim][Cl] + ultrasound
Pseudo-first-order model	R_e_ (fraction)	0.89817	0.92854	0.89419	0.93557
	k_1_ (min⁻^1^)	0.11248	0.13982	0.18495	0.23072
	R^2^	0.98423	0.97916	0.99344	0.99692
Pseudo-second-order model	R_e_ (fraction)	1.14939	1.14656	1.06291	1.06871
	k_2_ (min⁻^1^)	0.09044	0.12066	0.23728	0.26574
	R^2^	0.97458	0.96020	0.96272	0.98025

#### 3.5.2 Shrinking-core and diffusion-limited extraction models

Detailed model derivation is provided in the Supporting Information. As shown in Table 4, the extraction in different solvents and treatment systems follows an initial period of rapid growth and slow plateau. This pattern is consistent with several coupling steps, including solvent permeation into pores and oil films, oil film expansion and relaxation, interface separation, dissolution, and transport of dissolved components into the host liquid. The fitting platform recovery rate R_∞_ of [Bmim][Cl]-ultrasound combined treatment was the highest for all four solvents: toluene, p-xylene, m-xylene, and o-xylene were 95.2, 87.9, 92.4, and 88.9 wt%, respectively. The combined toluene system has the highest fitting platform recovery rate. This numerical model is consistent with the good solubility of toluene in heavy oil components, but only the fitted plateau values cannot determine the molecular source of solvent differences.

**Table 4:** Fitted parameters of mass-transfer kinetic models for heavy-oil extraction under different solvents and treatment conditions.

Solvent	Extraction system	R∞ (wt%)	kSCM (min⁻^1^)	SCM R^2^	SCM RMSE (wt%)	kD (min⁻^1^)	Fick R^2^	Fick RMSE (wt%)	km (min⁻^1^)	N–W R^2^	N–W RMSE (wt%)
Toluene	Solvent only	89.8	0.0319	0.977	4.40	0.00591	0.892	9.62	0.1124	0.984	3.66
Toluene	Ultrasound-assisted	90.8	0.0505	0.983	3.28	0.00970	0.941	6.08	0.1792	0.992	2.25
Toluene	[Bmim][Cl]-assisted	92.5	0.0401	0.979	4.47	0.00746	0.877	10.76	0.1406	0.977	4.65
Toluene	Combined [Bmim][Cl]–ultrasound	95.2	0.0618	0.981	3.37	0.01221	0.949	5.57	0.2219	0.995	1.73
p-xylene	Solvent only	82.3	0.0221	0.980	3.86	0.00423	0.875	9.70	0.0808	0.984	3.50
p-xylene	Ultrasound-assisted	83.7	0.0482	0.981	3.54	0.00903	0.904	7.95	0.1690	0.983	3.37
p-xylene	[Bmim][Cl]-assisted	85.6	0.0356	0.955	5.17	0.00657	0.945	5.71	0.1259	0.985	2.95
p-xylene	Combined [Bmim][Cl]–ultrasound	87.9	0.0477	0.948	5.36	0.00888	0.960	4.69	0.1692	0.980	3.30
m-xylene	Solvent only	87.0	0.0239	0.956	5.68	0.00458	0.909	8.17	0.0876	0.978	4.00
m-xylene	Ultrasound-assisted	88.4	0.0369	0.974	4.23	0.00687	0.937	6.58	0.1304	0.993	2.19
m-xylene	[Bmim][Cl]-assisted	90.4	0.0291	0.949	6.23	0.00543	0.920	7.85	0.1043	0.978	4.12
m-xylene	Combined [Bmim][Cl]–ultrasound	92.4	0.0468	0.962	5.27	0.00877	0.919	7.71	0.1656	0.978	4.07
o-xylene	Solvent only	84.0	0.0271	0.989	2.98	0.00517	0.875	10.11	0.0969	0.986	3.35
o-xylene	Ultrasound-assisted	84.9	0.0492	0.985	3.12	0.00932	0.906	7.77	0.1731	0.985	3.08
o-xylene	[Bmim][Cl]-assisted	86.8	0.0285	0.953	5.50	0.00533	0.943	6.07	0.1021	0.986	2.98
o-xylene	Combined [Bmim][Cl]–ultrasound	88.9	0.0643	0.963	4.49	0.01217	0.915	6.86	0.2277	0.976	3.65

Note: (1) R_∞_ is the fitted plateau heavy-oil recovery; (2) k_SCM_ is the apparent shrinking-core-model rate constant; (3) k_D_ is the lumped Fickian diffusion parameter; (4) k_m_ is the Noyes–Whitney liquid-film mass-transfer/dissolution rate constant; (5) SCM denotes the apparent shrinking-core model; (6) N–W denotes the Noyes–Whitney-type mass-transfer-controlled dissolution model; and (7) RMSE is the root-mean-square error between fitted and experimental extraction values.

After adding [Bmim] [Cl] or ultrasound, the fitted apparent rate parameters usually increase, and the combined system shows the maximum numerical increase. For example, in the toluene system, the apparent shrinkage core model rate constant k_SCM_ increased from 0.0319 min^−1^ to 0.0618 min⁻1, and the aggregated Fick's diffusion parameter k_D_ increased from 0.00591 min^−1^ to 0.01221 min ⁻^1^, Noyes Whitney type mass transfer/dissolution rate constant k_m_ increased from 0.1124 min^−1^ to 0.2219 min ⁻^1^. Combined with interface measurements, these changes are consistent with lower oil film peeling resistance and faster interface transport under combined treatment. The AFM trend is particularly applicable to hydrophobic SiO_2_ model interfaces. Ultrasonic waves can also thin the liquid film boundary layer and promote interface renewal through cavitation, acoustic flow, microjet, and local shear. Since these fitting parameters are empirical parameters, they do not isolate a single basic transmission step. In the evaluated models, the Noyce Whitney type liquid film mass transfer/dissolution model typically gives high R ^2^ values and low RMSE values. For the merged toluene system, the R^2^ is 0.995 and the RMSE is 1.73 wt%, which fits better numerically than the corresponding Fickian model. This result indicates that the Noyce Whitney equation provides the best empirical description for the dataset; This does not prove that liquid film mass transfer or interfacial dissolution is the only rate controlling step. The lower R^2^ and higher RMSE values of the Fickian model in several pure solvent systems also do not exclude internal diffusion. The apparent shrinking-core model also described most systems reasonably well, but the oil occurs as non-uniform films, pore-retained material, and resin–asphaltene aggregates rather than as an ideal spherical core. Accordingly, the model is used as an empirical representation of the progressive decrease in the extractable region, not as evidence of a strict geometric shrinking-core mechanism.

The three models provide complementary empirical descriptions of the multistep extraction process. The Noyes–Whitney-type model reflects liquid-film mass transfer and interfacial dissolution, the Fickian model represents diffusion-related contributions, and the apparent shrinking-core model describes the progressive decrease in the extractable region. The simultaneous increases in fitted plateau recovery and apparent rate parameters under the combined treatment are consistent with complementary interfacial regulation by [Bmim][Cl] and mass-transfer enhancement by ultrasound. However, the model fits do not uniquely assign the contribution of each physical step.

### 3.6 Molecular dynamics simulation

#### 3.6.1 MSD analysis

**Fig. 9:**
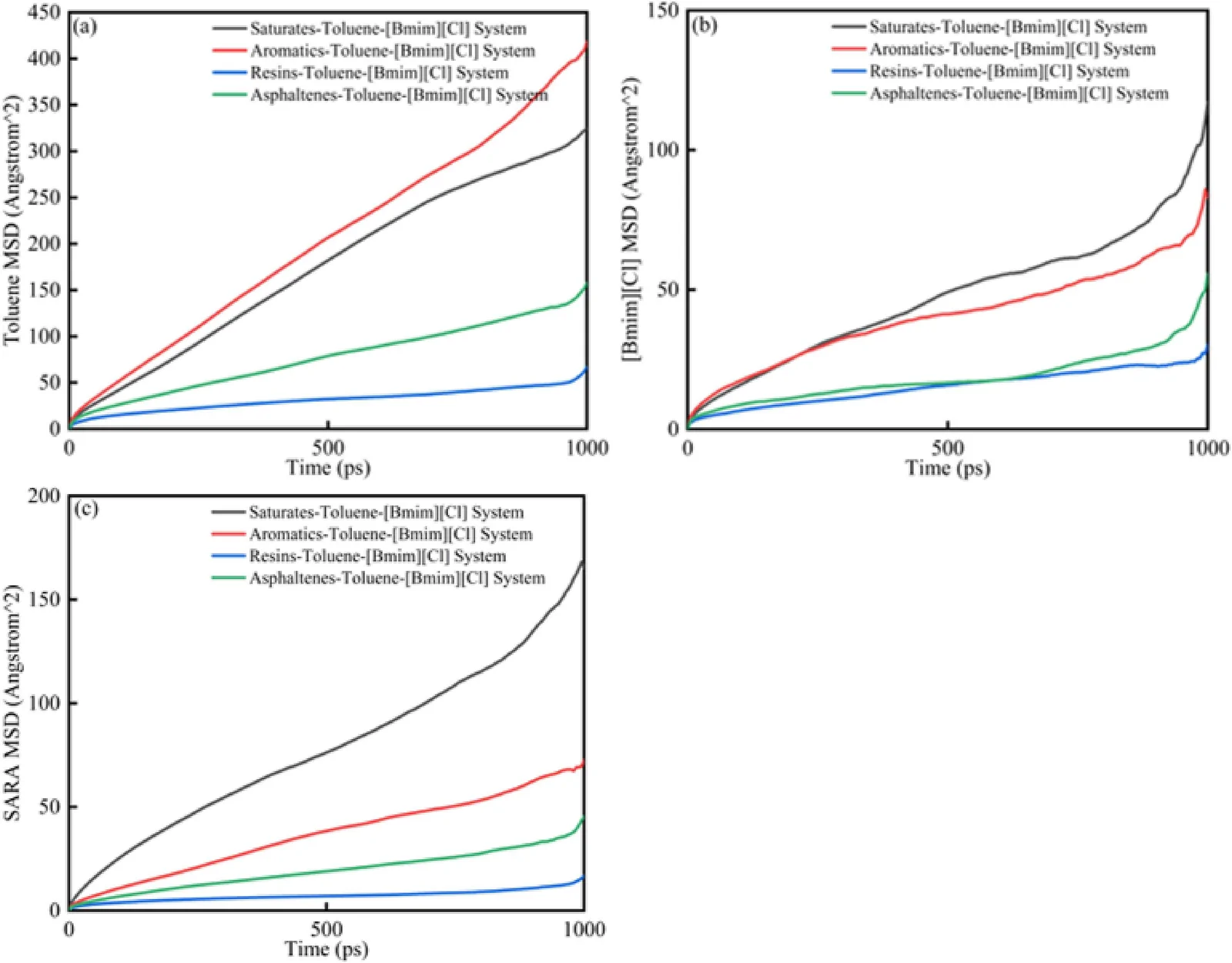
MSD profiles of (a) toluene, (b) [Bmim][Cl], and (c) SARA representative molecules in the SARA–toluene–[Bmim][Cl] systems.

Fig. 9 compares the MSD curves of toluene, [Bmim][Cl], and the SARA representative molecules in the toluene-based systems. Over the 1000 ps trajectories, the saturates and aromatics generally had larger MSD values than resins and asphaltenes. This ordering indicates greater relative mobility of the lighter components under the same simulation protocol. The lower MSD values of heavier molecules reflect their larger size and more persistent local associations with surrounding species. These observational results involve relative motion within the current finite model.

**Fig. 10:**
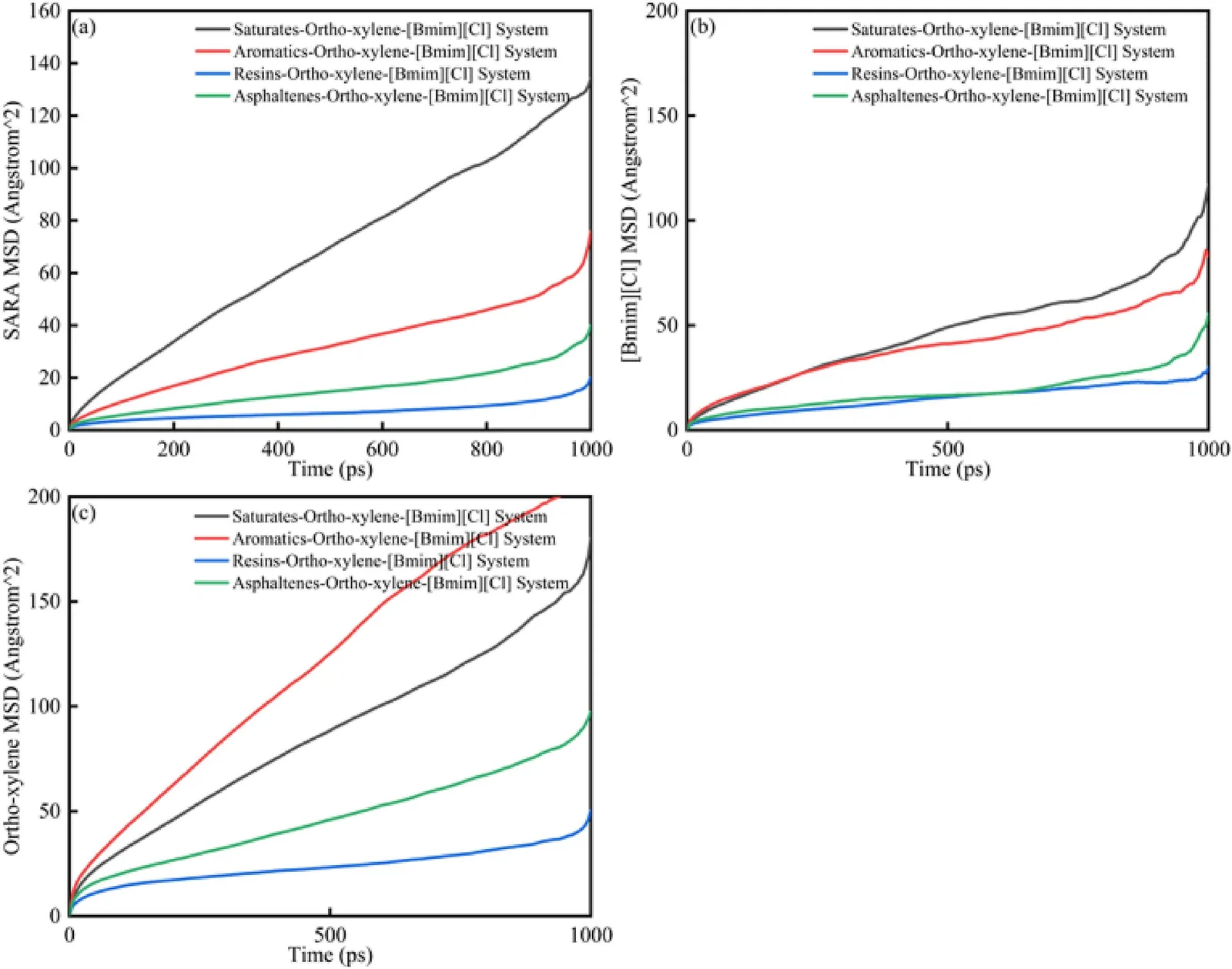
MSD profiles of (a) SARA representative molecules, (b) [Bmim][Cl], and (c) o-xylene in the SARA–o-xylene–[Bmim][Cl] systems.

Fig. 10 compares the relative molecular motion in the SARA-o-xylene −[Bmim] [Cl] system. The results were similar with the SARA-toluene-[Bmim][Cl] system, and.

[Bmim][Cl] follows a similar relative pattern in the four subsystems. In these models, lower MSD values in heavier molecular systems indicate stronger local limitations on molecular motion. This result is used for intra model comparison and does not establish a quantitative ranking for the bulk diffusion coefficient or solvent transport of o-xylene.

**Fig. 11:**
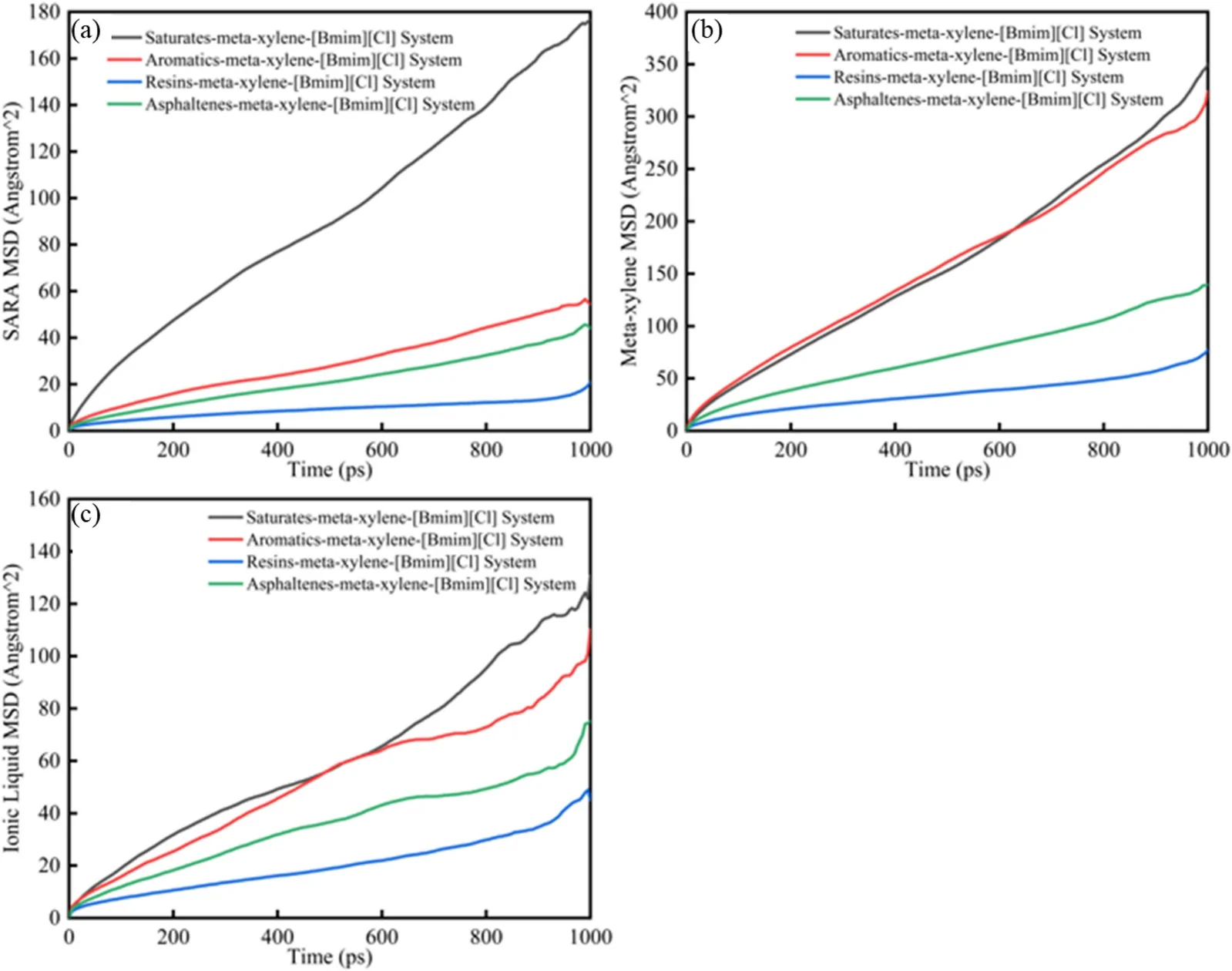
MSD profiles of (a) SARA representative molecules, (b) m-xylene, and (c) [Bmim][Cl] in the SARA–m-xylene–[Bmim][Cl] systems.

**Fig. 12:**
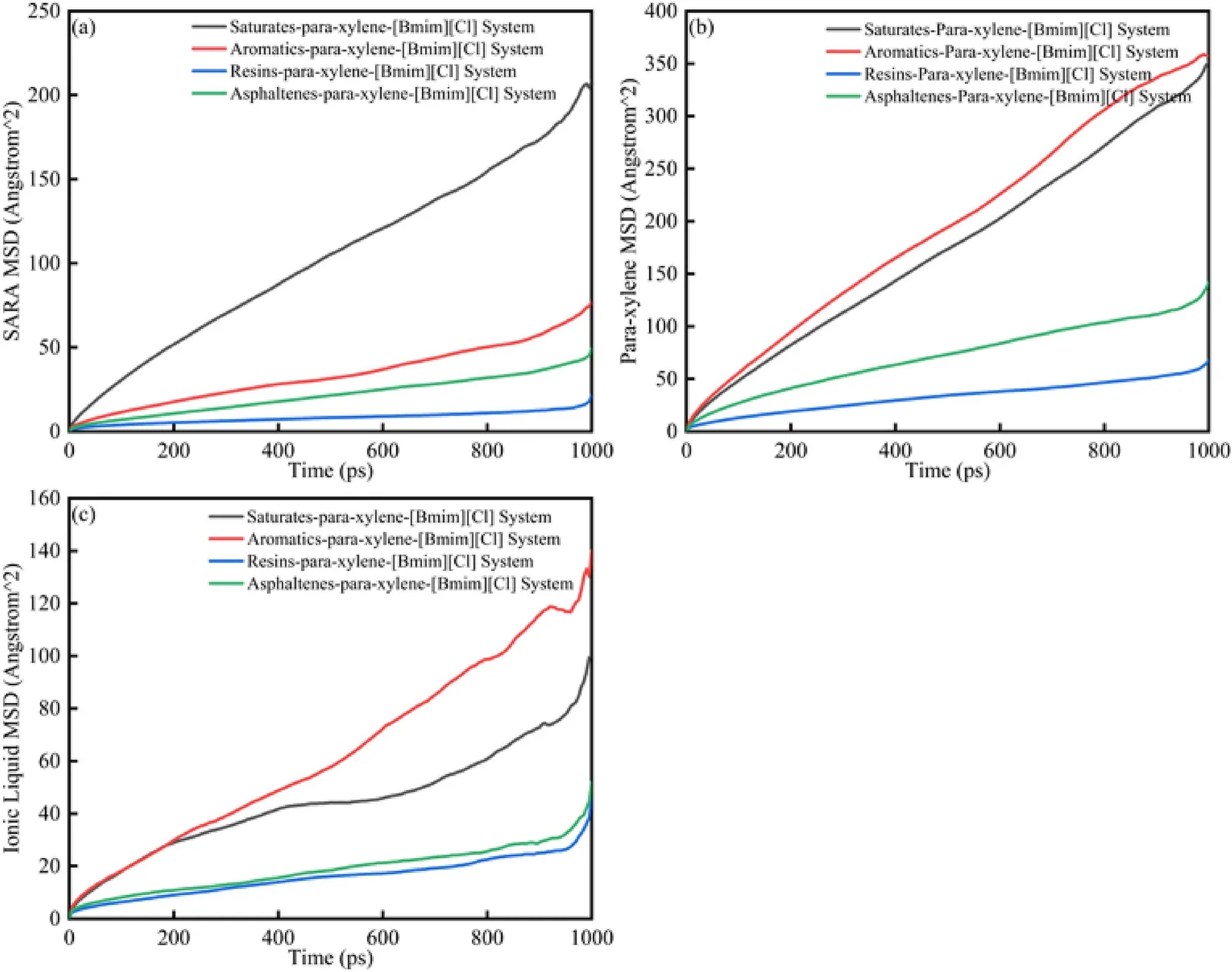
MSD profiles of (a) SARA representative molecules, (b) p-xylene, and (c) [Bmim][Cl] in the SARA–p-xylene–[Bmim][Cl] systems.

Fig. 11 showed that the saturates showed the highest MSD value than other three components in the SARA-m-xylene-[Bmim][Cl] simulation system, and then aromatics, resins and asphaltenes followed, which was due to the fact that saturates showed the lowest relative molecular mass and viscosity, and then other three components followed. In addition, the m-xylene showed higher MSD value than SARA and [Bmim][Cl] value under the same simulation systems. Fig. 12 compares the MSD curves of the SARA-p-xylene-[Bmim] [Cl] system, and the results were similar with Fig. 11.

Fig. 9, Fig. 10, Fig. 11, Fig. 12 show a roughly consistent relative order: saturates typically have the highest MSD values, followed by aromatics, while resins and asphaltenes move less within the simulated time window. Molecular size, aromatic structure, polarity, and local association all contribute to this pattern. A lower MSD does not necessarily mean weaker solvation; It reflect more enduring local connections. Therefore, MSD curves are used together with RDF and concentration distributions to compare the molecular behavior between models, rather than quantifying diffusion in bulk heavy oil. Because the simulation cells contain a limited number of SARA representative molecules and ionic-liquid ion pairs, the MSD-derived transport results may be affected by finite-size effects, limited molecular sampling, and trajectory-length dependence. Accordingly, these results are used only to compare relative molecular mobility trends among systems constructed using an identical simulation protocol and the results didn’t totally consistent with the statistically converged bulk-phase diffusion coefficients or as quantitative predictions of macroscopic heavy-oil transport.

#### 3.6.2 RDF analysis

##### 3.6.2.1 Solvent–SARA–[Bmim][Cl] RDF profiles

RDF analysis is used to describe the relative probability of distribution between different components within local spatial ranges, reflecting trends in local contact and association between molecules. It should be noted that RDF peak intensity does not directly equate to the strength of intermolecular interactions, especially in multicomponent systems where [Bmim][Cl] self-association, solvent dispersion, and local aggregation of representative SARA molecules are present simultaneously. To quantitatively compare the strength of different interactions, parameters such as the first coordination shell integral, coordination number, binding energy, or interaction energy must also be considered. The RDF results could compare local contact probabilities and spatial association trends among different components, rather than to quantitatively determine interaction strength. The RDF profiles for [Bmim][Cl], the SARA representative molecules, and the solvents are provided in Figs. S2–S5.

As shown in Fig. S2, the [Bmim][Cl]–[Bmim][Cl] curves in each RDF sub-chart show relatively high peaks in the short-range region, indicating that [Bmim][Cl] exhibits distinct local self-association and locally ordered structures in this simplified liquid-phase system. In contrast, the peak values of the component–[Bmim][Cl] curves are generally lower than those of the [Bmim][Cl]–[Bmim][Cl] curves. Across the SARA representatives, from the saturates and aromatics to the resins and asphaltenes, the SARA–[Bmim][Cl] curves show enhanced peak values and local contact characteristics in the short-range region, indicating that heavy representative molecules with aromatic structures and polar groups have a more pronounced local contact probability or association trend with [Bmim][Cl]. These profiles suggest local-microenvironment differences around the heavier representative molecules, but they do not quantify interaction strength or demonstrate disruption of real asphaltenes aggregates.

Fig. S3 shows the RDF curves for the o-xylene systems. [Bmim][Cl]–[Bmim][Cl] gives the most pronounced short-range peaks, indicating local ionic-liquid self-association in the finite cells. The asphaltenes–[Bmim][Cl] curves are more evident for the heavier representative molecules than saturates-[Bmim][Cl] in several subsystems, which was due to the relative changes in local contact. Fig. S4 presents the RDF curves for the m-xylene systems. The [Bmim][Cl]–[Bmim][Cl] curve remains dominant in the short-range region, while the component–[Bmim][Cl] curves vary among the four SARA subsystems. The more pronounced short-range features for several heavier representative molecules suggest differences in local association within the finite models. Because RDF peak height depends on particle selection and number density, these curves are used for comparison only and are not treated as binding-energy measurements. Fig. S5 shows the same general RDF pattern in the p-xylene systems. The ionic-liquid autocorrelation peaks dominate the short-range region, and the component–[Bmim][Cl] curves are more pronounced for resins and asphaltenes than for saturates subsystem.

##### 3.6.2.2 Peak-position and peak-intensity analysis

Similar trends were observed in the o-xylene, m-xylene, and p-xylene systems. The [Bmim][Cl] autocorrelation curves retained strong short-range peaks, showing that local ionic-liquid self-association was a prominent feature of these simplified liquid-phase models. The component–[Bmim][Cl] peaks for the resins and asphaltenes were generally more pronounced than those for the saturates, indicating differences in short-range contact probability. Because coordination numbers, first-shell integrals, and interaction energies were not calculated, these results are used only as qualitative evidence of local microenvironment differences.

The [Bmim][Cl] autocorrelation peaks were dominant in the simplified SARA–solvent–[Bmim][Cl] systems. The RDF is defined by Equations (17), (18).

(17) $$I_{\text{RDF}}=\frac{g_{\max,\text{component - IL}}}{g_{\max,\text{IL - IL}}}\times 100\%$$

(18) $$F_{\text{self}}=\frac{g_{\max,\text{IL - IL}}}{g_{\max,\text{component - IL}}}$$

From Table 5, the first [Bmim][Cl]–[Bmim][Cl] peaks (110.8–163.4) were much higher than the corresponding component–[Bmim][Cl] peaks (5.9–12.4) across the 16 models. The relative component–ionic-liquid peak intensities were 4.6–8.1% of the ionic-liquid autocorrelation peaks. Within the present models, these values indicate dominant local self-association of [Bmim][Cl] and weaker local component–[Bmim][Cl] correlations. They are comparative RDF descriptors, not quantitative measures of interaction energy.

**Table 5:** Comparative short-range RDF descriptors in the different SARA–solvent systems.

Solvent system	SARA component	First-peak position of [Bmim][Cl]–[Bmim][Cl] (Å)	g_max,IL-IL_	First-peak position of component–[Bmim][Cl] (Å)	g_max,_ _component-IL_	Relative RDF peak intensity, I_RDF_ (%)	Self-association dominance factor, F_self_
Toluene	Saturates	1.07	110.8	1.13	5.9	5.3	18.9
Toluene	Aromatics	1.02	146.0	1.02	6.7	4.6	21.8
Toluene	Resins	1.07	151.6	1.13	12.4	8.1	12.3
Toluene	Asphaltenes	1.13	146.0	1.13	7.9	5.4	18.6
o-xylene	Saturates	1.10	120.8	1.10	6.7	5.6	18.0
o-xylene	Aromatics	1.05	156.7	1.10	11.0	7.0	14.3
o-xylene	Resins	1.05	158.9	1.10	8.8	5.5	18.1
o-xylene	Asphaltenes	1.05	159.6	1.10	10.5	6.6	15.2
m-xylene	Saturates	1.09	123.4	1.09	7.6	6.2	16.2
m-xylene	Aromatics	1.09	158.6	1.14	9.2	5.8	17.3
m-xylene	Resins	1.09	162.3	1.09	10.4	6.4	15.6
m-xylene	Asphaltenes	1.09	156.2	1.14	9.2	5.9	17.1
p-xylene	Saturates	1.10	122.9	1.15	7.1	5.8	17.2
p-xylene	Aromatics	1.10	159.8	1.10	9.8	6.1	16.3
p-xylene	Resins	1.10	163.4	1.15	10.4	6.4	15.6
p-xylene	Asphaltenes	1.04	157.9	1.10	9.8	6.2	16.1

Averaged across the solvent models, the first component–[Bmim][Cl] peak values for the saturates, aromatics, resins and asphaltenes were 6.8, 9.2, 10.5, and 9.4, respectively. The corresponding mean relative RDF peak intensities were 5.7%, 5.9%, 6.6%, and 6.0%. The resins had the highest mean value, but the differences were small and the ordering was not consistent across solvents. For example, the toluene–resin peak was 12.4, numerically higher than those of the other toluene subsystems, whereas in o-xylene the aromatic peak (11.0) exceeded the resin peak (8.8). The mean asphaltenes value (6.0%) was only slightly higher than the aromatics value.

The [Bmim][Cl] autocorrelation peaks also varied among solvent systems. The lowest value was 110.8 in the toluene–saturates subsystem, whereas values above 160 occurred in several xylene–resins or xylene–aromatics subsystems. These differences reflect solvent geometry, local packing, and ionic arrangement. RDF peak height is influenced by particle selection, atoms, number density, and local structural order; it is not a direct measure of van der Waals or electrostatic interactions, binding free energy, or interaction energy. A higher first peak therefore indicates only a greater short-range probability for the selected particle pair.

Table 6 summarizes the averaged RDF descriptors for the SARA representative molecules. The values show dominant [Bmim][Cl] self-association and weaker component–[Bmim][Cl] correlations across the models. The resins had the highest mean local correlation, whereas the asphaltenes value did not consistently exceed that of aromatics. These descriptors are used only to compare local-contact trends.

**Table 6:** Averaged RDF descriptors for the SARA representative molecules.

SARA component	Mean g_max,IL-IL_	Mean g_max, component-IL_	Mean relative RDF peak intensity, I_RDF_ (%)	Mean self-association dominance factor, F_self_	Relative local-association tendency
Saturates	119.5	6.8	5.7	17.6	Weakest
Aromatics	155.3	9.2	5.9	17.4	Moderate
Resins	159.1	10.5	6.6	15.2	Relatively highest
Asphaltenes	154.9	9.4	6.0	16.8	Moderate to relatively high

#### 3.6.3 Concentration analysis

##### 3.6.3.1 Concentration profiles

To compare the spatial distributions of the SARA representative molecules, aromatic solvents, and [Bmim][Cl], concentration profiles were examined for each simulation box. These profiles visualize relative distributions under the same simulation protocol. The profiles are used as qualitative molecular-scale descriptors of local distribution differences.

**Fig. 13:**
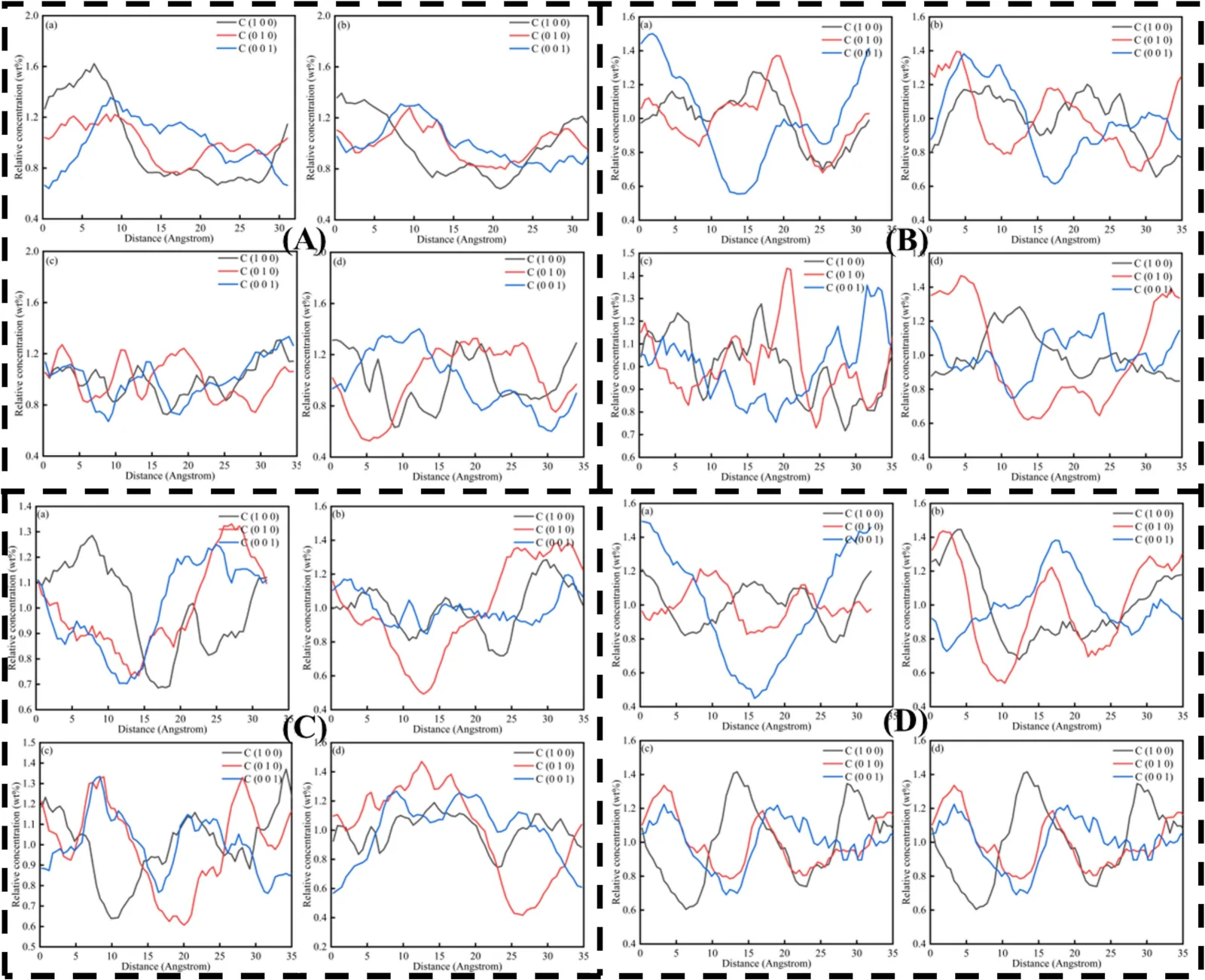
SARA concentration (wt%) in different simulation systems:(A) Toluene-SARA-[Bmim][Cl]; (B) o-Xylene-SARA-[Bmim][Cl]; (C) m-Xylene-SARA-[Bmim][Cl]; (D) p-Xylene-SARA-[Bmim][Cl]. (a) Saturates; (b) Aromatics;(c) Resins; (d) Asphaltenes.

Fig. 13 compares the concentration profiles of the SARA molecules in the solvents systems. The profiles differed among systems, and the resins and asphaltenes showed greater local heterogeneity in several models than saturates and aromatics. These differences reflect molecular size, aromaticity, polarity, and finite-cell packing. Without regional enrichment factors or partition coefficients, however, they are not treated as quantitative evidence of a solvent-structure effect.

**Fig. 14:**
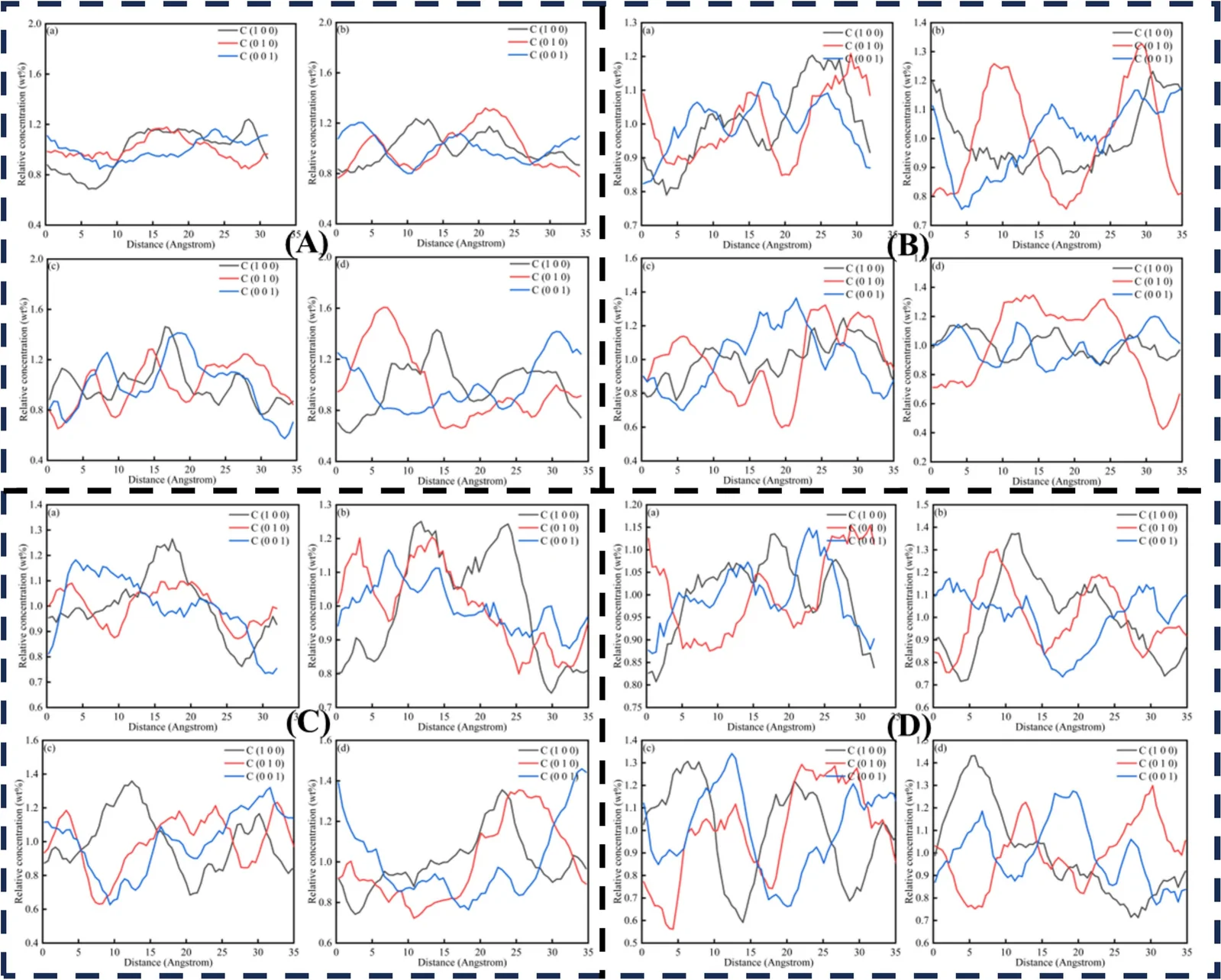
Solvent concentration (wt%) analysis of different simulation systems: (A) Toluene-SARA-[Bmim][Cl] system; (B) o-xylene-SARA-[Bmim][Cl] system; (C) m-xylene-SARA-[Bmim][Cl] system; (D) p-xylene-SARA-[Bmim][Cl] system. (a) Saturates; (b) Aromatics; (c) Resins; (d) Asphaltenes.

Fig. 14 compares the solvent concentration profiles across the simulation systems. The profiles for toluene, o-xylene, m-xylene, and p-xylene differed in shape, indicating solvent-dependent local distributions within the finite cells. Due to the simulation limitation, the profiles are used only for qualitative comparison and do not establish a quantitative solvent-structure effect.

**Fig. 15:**
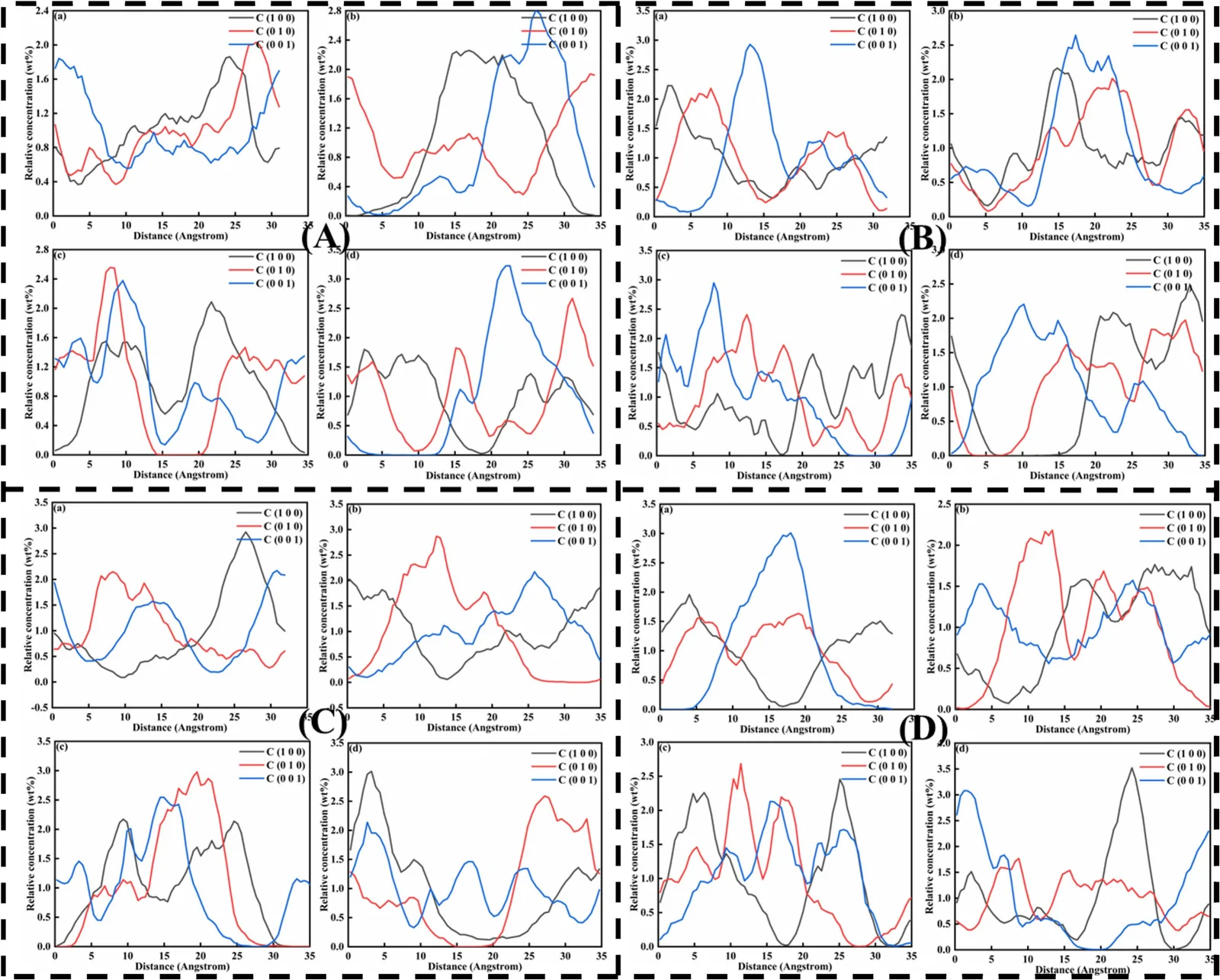
[Bmim][Cl] concentration analysis of different simulation systems: (A) Toluene-SARA-[Bmim][Cl] system; (B) o-xylene-SARA-[Bmim][Cl] system; (C) m-xylene-SARA-[Bmim][Cl] system; (D) p-xylene-SARA-[Bmim][Cl] system. (a) Saturates; (b) Aromatics; (c) Resins; (d) Asphaltenes.

Fig. 15 showed the [Bmim][Cl] concentration profiles in the solvents-SARA-[Bmim][Cl] systems. Their shapes differed among the finite liquid-phase models, suggesting that the solvent environment may influence the local distribution of [Bmim][Cl]. Fig. 15 showed that the [Bmim][Cl] distributed evenly than SARA and solvents. Because the models contain no explicit interface and no regional integration was performed, the profiles do not quantify interfacial enrichment, equilibrium partitioning, or solvent selectivity.

##### 3.6.3.2 Comparative analysis of concentration distributions

The concentration profiles showed SARA, solvents and [Bmim][Cl] dependent local differences within the simplified liquid-phase models. The overall average concentration in the three directions is defined in **Equation** (19):

(19) $${\overset{¯}{C}}_{i}=\frac{1}{3L}\sum_{\mathrm{hkl}=(100),(010),(001)}\int_{0}^{L}C_{i,hkl}\left(z\right),dz$$

The maximum local enrichment factor is defined in **Equation** (20):

(20) $$E_{\max,i}=\frac{C_{\max,i}}{{\overset{¯}{C}}_{i}}$$

The spatial non-uniformity index is defined in **Equation** (21):

(21) $$H_{i}=\frac{C_{\max,i}-C_{\min,i}}{{\overset{¯}{C}}_{i}}$$

The directional distribution ratio is defined in **Equation** (22):

(22) $$K_{\text{dir},i}=\frac{\max({\overset{¯}{C}}_{100},{\overset{¯}{C}}_{010},{\overset{¯}{C}}_{001})}{\min({\overset{¯}{C}}_{100},{\overset{¯}{C}}_{010},{\overset{¯}{C}}_{001})}$$

E_max_ reflects the degree of increase in local concentration peaks relative to the overall average concentration, H reflects the peak-valley fluctuation amplitude in the entire simulation box, and K_dir_ is used to compare the average distribution differences among the three statistical directions. In the simplified representative-molecule systems, [Bmim][Cl] showed more pronounced local association with resins and asphaltenes than with saturates. Because no coordination-number or interaction-energy analysis was performed and no polydisperse asphaltene aggregate was modeled, these profiles are used only to compare local distribution trends.

Table 7 shows that the overall mean normalized concentrations of the SARA molecules were close to 1, as expected from the normalization procedure; this agreement is not an independent mass-conservation test. The local peak factor, E_max, SARA_, ranged from 1.31 to 1.60, and the spatial non-uniformity index, H_SARA_, ranged from 0.63 to 1.02. The largest E_max,SARA_ value (1.60) occurred in the toluene–saturates subsystem. The largest H_SARA_ values (1.02) occurred in the m-xylene–asphaltenes and p-xylene–saturates subsystems, indicating comparatively pronounced local fluctuations. In contrast, the toluene–resins, m-xylene–saturates, and o-xylene–resins systems were less heterogeneous. Values of K_dir, SARA_ ranged only from 1.000 to 1.030, indicating little difference among the integrated directional averages.

**Table 7:** Comparative descriptors extracted from the concentration profiles.

Solvent	SARA component	Normalized SARA concentration	E_max,SARA_	H_SARA_	K_dir,SARA_	Normalized solvent concentration	E_max,solvent_	H_solvent_	K_dir,solvent_	Normalized [Bmim][Cl] concentration	E_max,IL_	H_IL_	K_dir,IL_
Toluene	Saturates	0.65–1.60	1.60	0.95	1.004	0.68–1.22	1.23	0.55	1.017	0.37–2.03	1.97	1.61	1.099
Toluene	Aromatics	0.66–1.39	1.39	0.73	1.024	0.79–1.31	1.30	0.52	1.003	0.08–2.62	2.60	2.52	1.017
Toluene	Resins	0.70–1.33	1.33	0.63	1.007	0.60–1.45	1.44	0.85	1.009	0.08–2.56	2.50	2.43	1.013
Toluene	Asphaltenes	0.53–1.38	1.39	0.86	1.003	0.62–1.60	1.61	0.98	1.008	0.09–3.21	3.13	3.04	1.008
o-xylene	Saturates	0.55–1.50	1.49	0.94	1.029	0.81–1.19	1.19	0.39	1.015	0.11–2.87	2.92	2.81	1.089
o-xylene	Aromatics	0.62–1.39	1.39	0.77	1.014	0.77–1.32	1.32	0.55	1.013	0.09–2.56	2.56	2.47	1.006
o-xylene	Resins	0.74–1.41	1.41	0.67	1.002	0.61–1.33	1.33	0.73	1.004	0.10–2.81	2.86	2.76	1.045
o-xylene	Asphaltenes	0.63–1.46	1.46	0.83	1.010	0.43–1.33	1.33	0.90	1.010	0.09–2.27	2.24	2.16	1.022
m-xylene	Saturates	0.68–1.32	1.31	0.63	1.000	0.74–1.25	1.26	0.52	1.014	0.07–2.53	2.54	2.46	1.114
m-xylene	Aromatics	0.49–1.37	1.38	0.88	1.006	0.74–1.24	1.25	0.50	1.004	0.02–2.81	2.77	2.75	1.021
m-xylene	Resins	0.61–1.33	1.33	0.72	1.014	0.64–1.35	1.35	0.71	1.012	0.10–2.95	2.78	2.68	1.048
m-xylene	Asphaltenes	0.43–1.46	1.44	1.02	1.011	0.74–1.45	1.45	0.71	1.011	0.10–2.80	2.76	2.66	1.020
p-xylene	Saturates	0.46–1.48	1.47	1.02	1.030	0.81–1.14	1.15	0.33	1.017	0.10–3.00	2.98	2.88	1.050
p-xylene	Aromatics	0.55–1.42	1.43	0.88	1.011	0.72–1.37	1.37	0.65	1.011	0.07–2.14	2.09	2.02	1.032
p-xylene	Resins	0.61–1.40	1.41	0.80	1.004	0.58–1.33	1.33	0.75	1.009	0.09–2.53	2.39	2.31	1.035
p-xylene	Asphaltenes	0.61–1.40	1.42	0.80	1.002	0.73–1.37	1.36	0.63	1.010	0.12–3.12	3.09	2.97	1.066

Note: Concentration ranges were calculated from the x-, y-, and z-direction profiles. Emax is a local peak factor rather than an interfacial thermodynamic enrichment factor. Kdir is a directional-distribution ratio and should not be interpreted as an equilibrium partition coefficient between independent phases.

For the solvent profiles, E_max,solvent_ ranged from 1.15 to 1.61 and H_solvent_ ranged from 0.33 to 0.98. In the toluene–asphaltenes system, the normalized solvent concentration ranged from 0.62 to 1.60, and the E_max,solvent_ value was 1.61, H_solvent_ value was 0.98, H_solvent_ was also relatively high (0.85) in the toluene–resins system. By contrast, the p-xylene–saturates subsystem ranged from 0.81 to 1.14, and the H_solvent_ value was 0.33, indicating a comparatively uniform solvent profile. The o-xylene and m-xylene systems were generally intermediate. K_dir,solvent_ value ranged from 1.003 to 1.017, indicating that the observed differences mainly reflected local fluctuations rather than stable directional partitioning.

The [Bmim][Cl] concentration profiles showed greater local heterogeneity than the SARA-component and solvents profiles. The maximum local peak factor of [Bmim][Cl] ranged from 1.97 to 3.13, and the spatial non-uniformity index ranged from 1.61 to 3.04. For example, the normalized [Bmim][Cl] concentration in the toluene–asphaltene system ranged from 0.09 to 3.21 (E_max,IL_ = 3.13, H_IL_ = 3.04). These profile fluctuations are consistent with pronounced local [Bmim][Cl] self-association in the finite liquid-phase models; they do not demonstrate interfacial enrichment or an equilibrium partition coefficient. However, K_dir,IL_ ranged from 1.006 to 1.114. The integrated directional averages differed only modestly over the 0–35 Å range. The concentration profiles showed solvents and component-dependent local fluctuations, rather than a stable directional partitioning of [Bmim][Cl].

#### 3.6.4 Temperature and energy trajectory checks

**Fig. 16:**
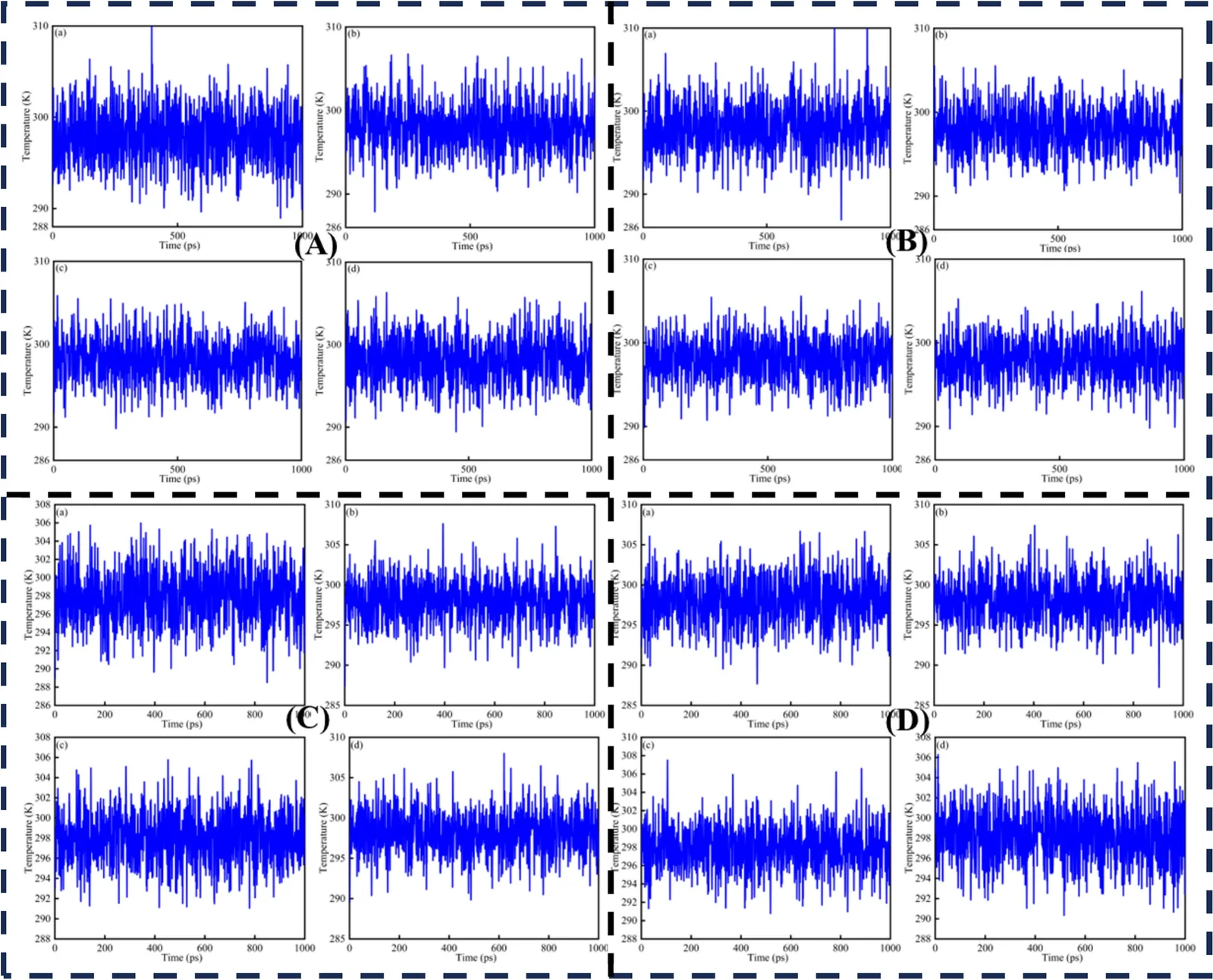
Temperature balance analysis of different simulation systems: (A) Toluene-SARA-[Bmim][Cl] system; (B) o-xylene-SARA-[Bmim][Cl] system; (C) m-xylene-SARA-[Bmim][Cl] system; (D) p-xylene-SARA-[Bmim][Cl] system. (a) Saturates; (b) Aromatics; (c) Resins; (d) Asphaltenes.

The temperature–time profiles in Fig. 16 were used to check whether the thermostat maintained the target temperature during the trajectories. All systems were simulated at 298 K, and the temperatures fluctuated around this value throughout the 1000 ps runs. The profiles provide a basic check of numerical stability within the simulated time window. Fig. 16 indicated that the all the simulation systems reached temperature equilibrium when the simulation reached 1000 ps.

**Fig. 17:**
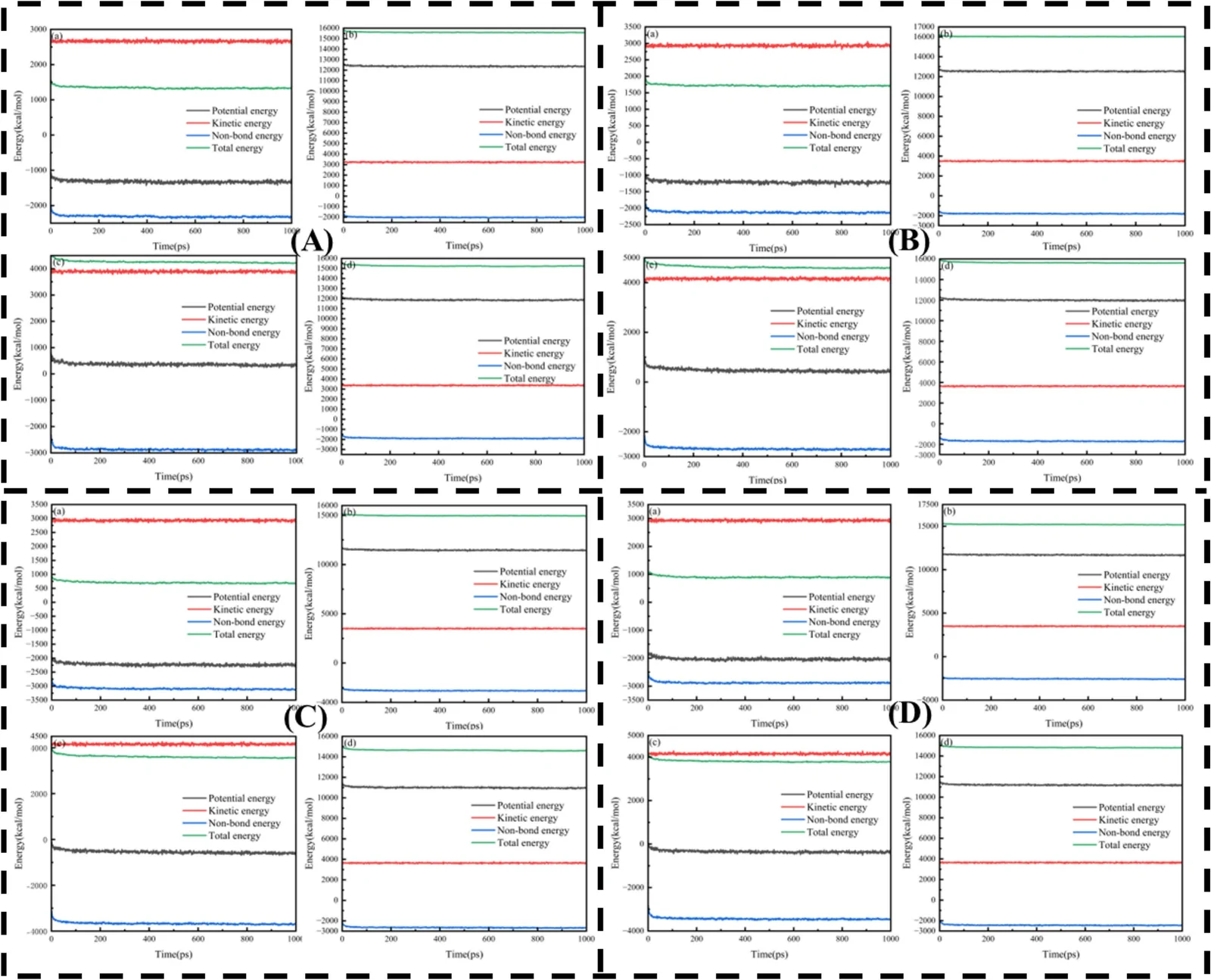
Energy balance analysis of different simulation systems: (A) Toluene-SARA-[Bmim][Cl] system; (B) o-xylene-SARA-[Bmim][Cl] system; (C) m-xylene-SARA-[Bmim][Cl] system; (D) p-xylene-SARA-[Bmim][Cl] system. (a) Saturates; (b) Aromatics; (c) Resins; (d) Asphaltenes.

The energy–time curves in Fig. 17 changed more strongly during the early part of each trajectory and then fluctuated within narrower ranges. This behavior indicates that the energy traces were comparatively stable over the later part of the 1000 ps runs.

#### 3.6.5 Conformational analysis

**Fig. 18:**
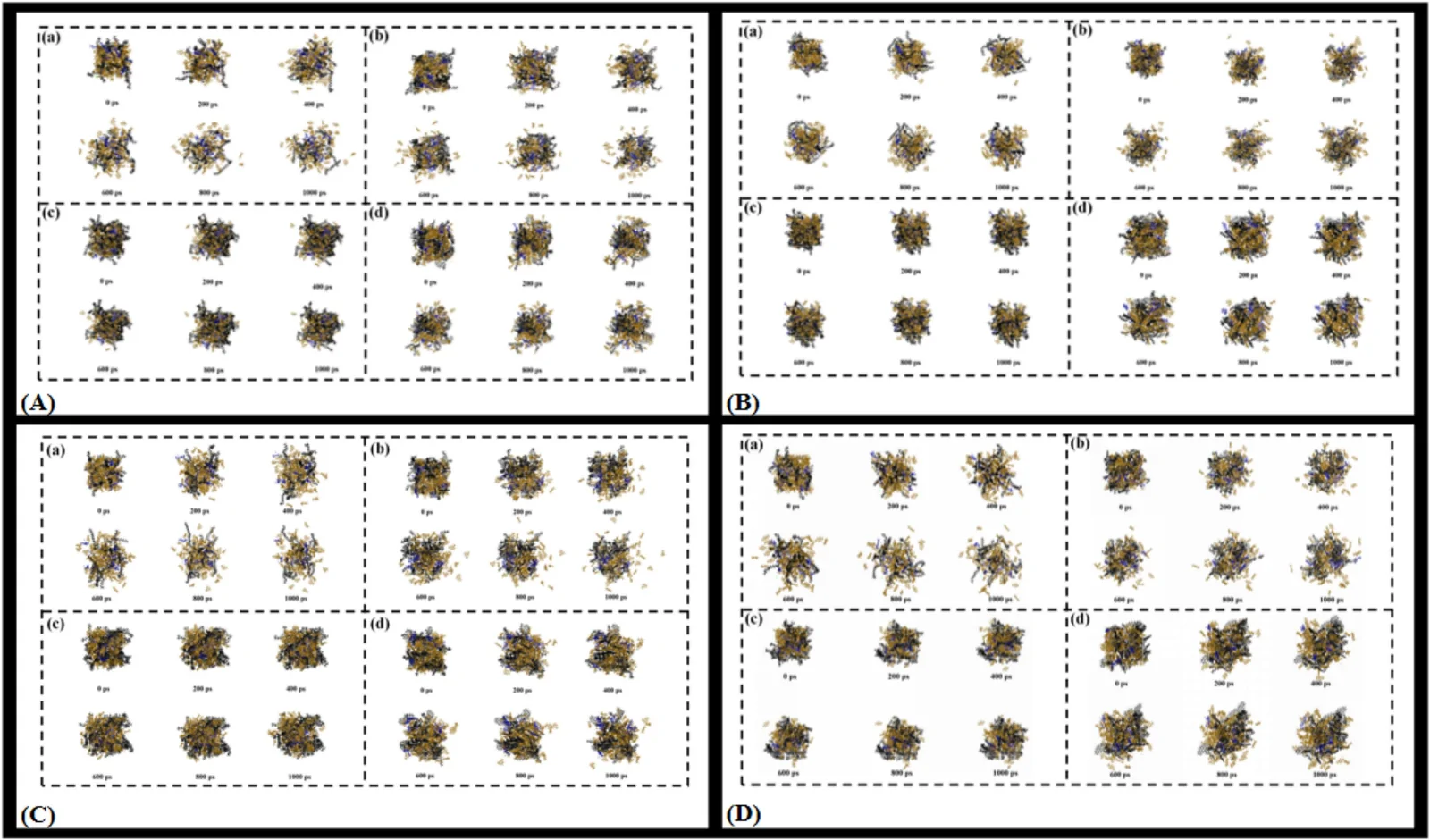
Configurational snapshots of the (A) toluene, (B) o-xylene, (C) m-xylene, and (D) p-xylene SARA–[Bmim][Cl] systems: (a) saturates, (b) aromatics, (c) resins, and (d) asphaltenes.

In the current trajectory, saturates and aromatics typically exhibit greater relative displacement than resins and asphaltenes. There are also differences among the four solvent models, but not every component has the same order. Due to the simulation containing a uniform periodic liquid phase without clear mineral or oil–water interfaces, they do not support conclusions about interface stratification or preferential ion orientation. Therefore, Fig. 18 is only used for qualitative comparison of local configurations within finite model elements.

Fig. 18 shows a snapshot of the conformational evolution of representative SARA molecules and [Bmim] [Cl]. These images allow for visual comparison of dispersion and local clustering within a simulated time window. The snapshot in Fig. 18 shows that representative SARA molecules adopt different local arrangements around [Bmim] [Cl] during the trajectory process. Resins and asphaltenes aggregate more locally in several frameworks, while saturates and aromatics are typically more dispersed. These visual differences may reflect molecular size, aromatic structure, polarity, and local contact with ionic liquid species. Due to the fact that cells only contain a limited number of representative molecules, snapshots are only used to compare qualitative conformational trends between solvent models. The comparative conformational analysis is provided in the Supporting Information, and Table S4 lists the corresponding illustrative trajectory descriptors.

#### 3.6.6 Combined analysis of experiments and molecular dynamics simulations

The experimental observations and MD descriptors showed qualitative correspondence in some trends, while other results differed. These comparisons are discussed in the Supporting Information and summarized in Table S5. They are not treated as quantitative validation of the experimental results.

### 3.7 [bmim][cl] and ultrasound combination mechanism

**Fig. 19:**
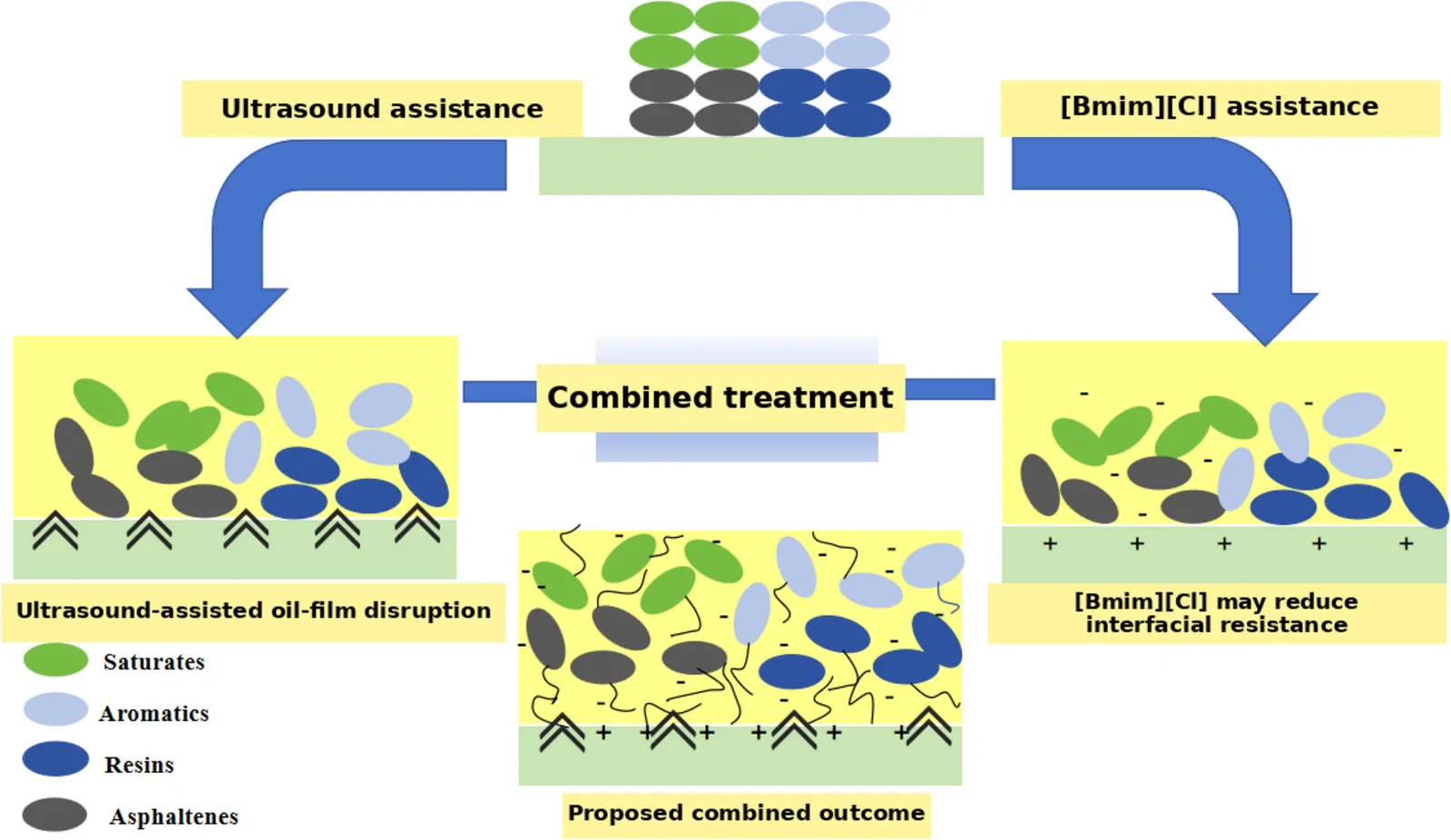
Proposed complementary roles of [Bmim][Cl] and ultrasound in heavy oil–solid separation.

Under non-sonicated conditions, [Bmim][Cl] is proposed to promote heavy-oil release primarily through interfacial regulation. The lower interfacial tension, more water-wet post-treatment solids, and reduced normalized AFM force at the hydrophobized SiO_2_ model interface are consistent with lower resistance to oil-film detachment. The homogeneous liquid-phase MD models further show short-range association between [Bmim][Cl] and resins or asphaltenes, but they do not simulate enrichment at a mineral interface. Ultrasound can enhance oil-film disruption, interfacial renewal, and mass transfer through cavitation, acoustic streaming, microjets, and local shear. In the combined system, [Bmim][Cl] may reduce interfacial resistance, while ultrasound accelerates transport and disruption of the destabilized oil film. This proposed mechanism is illustrated schematically in Fig. 19.

**Fig. 20:**
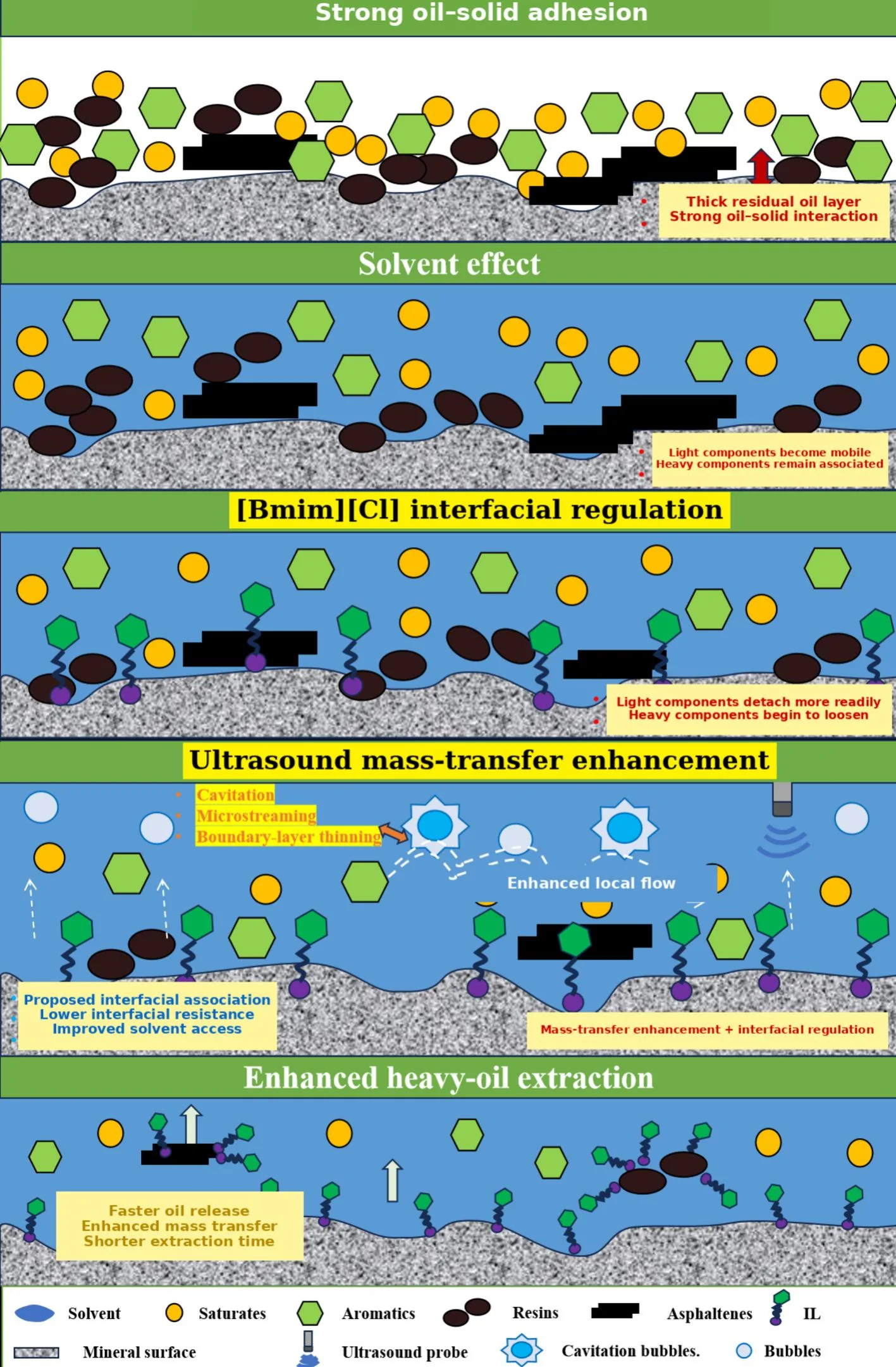
Schematic illustration of the mechanism of heavy oil–solid separation under combined ultrasound and [Bmim][Cl] assistance.

As shown in Fig. 20, comprehensive test results of heavy oil recovery efficiency, SARA composition, extraction kinetics, contact angle, interfacial tension, zeta potential, and AFM measurements at the hydrophobized SiO_2_ model oil–solid interface indicate that the improved performance under the combined ultrasound–[Bmim][Cl] treatment can be interpreted in terms of complementary mass-transfer enhancement and interfacial regulation. Ultrasonic cavitation, acoustic streaming, and local shear can promote solvent entry into oil-containing solid pores, disturb residual oil films, and accelerate the migration of the oil phase to the bulk solvent phase; the lower interfacial tension and normalized model-interface force are consistent with [Bmim][Cl] reducing resistance to oil-film detachment. Meanwhile, the SARA results and the qualitative MD comparisons suggest a more pronounced local contact trend between [Bmim][Cl] and resins or asphaltenes molecules containing aromatic structures and polar groups. This model-level trend may help interpret the observed small changes in recovered-oil SARA mass fractions, but it is not a quantitative simulation of their release from natural oil-sand surfaces. Therefore, the improved performance under the combined treatment was due to the fact that ultrasound promoted mass transfer and interfacial renewal, and [Bmim][Cl] reduced interfacial resistance and regulated the behavior of heavy components. Statistically significant synergy was observed for the toluene system (p = 0.023). The p-xylene system showed a borderline statistically significant positive interaction (p = 0.049), whereas the m-xylene and o-xylene systems showed positive but statistically non-significant interaction estimates (p = 0.198 and 0.158, respectively). It should be noted that the AFM and molecular dynamics models used in this paper are simplified. The AFM measurements use a hydrophobized SiO_2_ model oil–solid interface that does not reproduce the mineralogical composition, surface morphology and roughness, or wetting heterogeneity of natural oil sands; the force values are therefore used for controlled comparison among treatments within this model and are not interpreted as direct quantitative adhesion forces for natural oil-sand surfaces. The MD simulations use SARA representative molecules and periodic liquid-phase models. Accordingly, these model-based results support mechanistic trend interpretation rather than direct quantitative reproduction of the complex interfaces and ultrasonic cavitation processes of natural oil sands.

## 4 Conclusion

This study systematically examined combined ultrasound–ionic-liquid-assisted solvent extraction for heavy oil–solid separation. Four treatment modes were compared: solvent only, ultrasound-assisted extraction, [Bmim][Cl]-assisted extraction, and combined [Bmim][Cl]–ultrasound extraction. The experiment results included recovery, viscosity, C/H ratio, SARA composition, wettability, interfacial tension, normalized AFM interaction force at the hydrophobized SiO_2_ model interface, and zeta potential. In addition, the molecular dynamics simulation was conducted to study the ultrasound–ionic-liquid-assisted solvent extraction mechanism from molecular perspective.

Statistical analysis shows that toluene has a significant synergistic interaction (p = 0.023), while p-xylene has a critical statistically significant positive interaction (p = 0.049); The interaction between m-xylene and o-xylene is positive, but not statistically significant (p = 0.198 and 0.158, respectively). Among all testing systems, joint processing resulted in the highest average recovery rate and was associated with lower interfacial tension, lower normalized model interfacial force, and more wet post-treatment solids. The experimental trends are consistent with ultrasound promoting solvent penetration, interfacial renewal, and disruption of residual oil films, and with [Bmim][Cl] modifying interfacial properties, wettability, and the normalized force at the model interface. Under the combined treatment, heavy-oil migration was accelerated and small changes occurred in the resin and asphaltene mass fractions of the recovered oil.

Molecular dynamics simulations are used to compare the local behavior of [Bmim] [Cl] and SARA representative molecules in four solvent environments, providing molecular scale explanations for selected experimental trends. The experimental characterization and simulation together constitute the framework for examining solvent ionic liquid ultrasonic assisted heavy oil solid separation. The molecular dynamics simulation results provide qualitative trend explanations for experimental phenomena at the molecular scale. In the simplified SARA representative molecular model used in this paper, [Bmim][Cl] exhibits relatively more pronounced local contact and association trends with resins and asphaltenes representative molecules containing aromatic structures and polar groups, while local association with saturates is relatively weak. This trend is considered only as qualitative context for the small changes in recovered-oil resins and asphaltenes mass fractions.

It should be emphasized that this paper does not establish a quantitative correlation between simulation and experimental indicators, such as an R^2^ relationship between MSD-derived relative mobility and experimental recovery efficiency. Therefore, the relationship between MD results and experimental results should be understood as qualitative trend alignment, rather than strict quantitative correlation. Since this paper uses simplified representative SARA molecules and a limited-size periodic liquid-phase model, and does not directly simulate real mineral surfaces, aged oil films, pore mass transfer processes, or ultrasonic cavitation, the MD results are not used to quantitatively predict macroscopic recovery efficiency, real oil–sand interfacial desorption energy, or actual mass transfer rates. Therefore, the MD simulation in this paper should be understood as a simplified molecular-scale analysis of local distributions and associations among [Bmim][Cl], aromatic solvents, and SARA representative molecules. Specifically, in the simplified homogeneous liquid-phase models, resins and asphaltenes showed identifiable local contact and association trends with [Bmim][Cl], although the magnitude varied among the solvent systems. These results provide qualitative molecular-scale support for explaining the changes in recovered-oil heavy-component composition and interfacial behavior observed experimentally, but do not directly simulate mineral surface desorption or real oil-film detachment processes in natural oil sands.

Funding.

This project was supported by Hainan Provincial Natural Science Foundation of China (526MS0082, 224QN187), Hainan University Startup Fund (XJ2300004011), Open Project Program of Shandong Engineering Research Center for Environmental Protection and Remediation on Groundwater (801KF2025-6), Hainan Province Graduate Innovation Research Project (Qhys2024-104), Experimental Teaching Center for Basic Chemistry, School of Chemistry and Chemical Engineering, Hainan University (S202510589135, SA2500002086).

## CRediT authorship contribution statement

**Liping Zeng:** Writing – review & editing, Writing – original draft, Resources, Methodology, Formal analysis, Conceptualization. **Xuanrui Wang:** Project administration, Methodology, Data curation. **Ke Xu:** Writing – original draft, Software, Methodology. **Jinze Du:** Writing – review & editing, Resources, Funding acquisition, Formal analysis. **Yuxing Tan:** Writing – original draft, Visualization, Supervision, Software. **Pindong Tan:** Visualization, Software, Formal analysis, Data curation. **Yu Sun:** Visualization, Data curation, Conceptualization. **Jinjian Hou:** Writing – review & editing, Software, Investigation, Funding acquisition.

## Declaration of competing interest

The authors declare that they have no known competing financial interests or personal relationships that could have appeared to influence the work reported in this paper.

## Appendix A Supplementary data

Supplementary Data 1 The supplementary material was Supporting Information.
