# Supplementary material for: Combined effects of ultrasound and [bmim][cl] on heavy oil–solid separation: experiments and molecular dynamics simulations
Source: Ultrason Sonochem. 2026 Aug 22;133:108019. doi: 10.1016/j.ultsonch.2026.108019 (PMC13543103; doi:10.1016/j.ultsonch.2026.108019)
Supplement: Supplementary file 1 [file mmc1.docx]

**Combined Effects of Ultrasound and [Bmim][Cl] on Heavy Oil–Solid Separation: Experiments and Molecular Dynamics Simulations**

Liping Zeng^1#^, Xuanrui Wang^1#^, Ke Xu^1,2#^, Jinze Du^3^, Yuxing Tan^1^,

Pindong Tan^1^, Yu Sun^4^, Jinjian Hou^1*^

^1^School of Chemistry and Chemical Engineering, Hainan University, Haikou 570228, China

^2^School of Chemical Engineering, Ocean and Life Sciences, Dalian University of Technology, Panjin 124221, China

^3^Zhejiang Institute of Tianjin University, Ningbo 315000, China

^4^School of Chemical Engineering, Tianjin University, Tianjin 300072, China

Corresponding Author(*): Jinjian Hou (houjinjianhn@163.com)

Note (#): The three authors contributed equally.

**Funding**.

This project was supported by Hainan Provincial Natural Science Foundation of China (526MS0082, 224QN187), Hainan University Startup Fund (XJ2300004011), Open Project Program of Shandong Engineering Research Center for Environmental Protection and Remediation on Groundwater (801KF2025-6), Hainan Province Graduate Innovation Research Project (Qhys2024-104), Experimental Teaching Center for Basic Chemistry, School of Chemistry and Chemical Engineering, Hainan University (S202510589135, SA2500002086).

**Figure S1.** Overview of the experimental and molecular-simulation workflow

**Figure S2.** RDF profiles for the toluene–SARA–[Bmim][Cl] systems: (a) saturates, (b) aromatics, (c) resins, and (d) asphaltenes.

**Figure S3.** RDF profiles for the o-xylene–SARA–[Bmim][Cl] systems: (a) saturates, (b) aromatics, (c) resins, and (d) asphaltenes.

**Figure S4.** RDF profiles for the m-xylene–SARA–[Bmim][Cl] systems: (a) saturates, (b) aromatics, (c) resins, and (d) asphaltenes.

**Figure S5.** RDF profiles for the p-xylene–SARA–[Bmim][Cl] systems: (a) saturates, (b) aromatics, (c) resins, and (d) asphaltenes.

**Table S1.** Experimental conditions

| Experiment section | Solvent | Oil-sand sample (g) | Solvent  (mL) | [Bmim][Cl] (mL) | Ultrasound conditions | Extraction time (min) | Temperature (°C) | Post-processing |
| --- | --- | --- | --- | --- | --- | --- | --- | --- |
| 2.2.1 | Toluene,  o-xylene,  m-xylene,  p-xylene | 5.0 | 30 | / | / | 60 | 30 | Centrifugation (5 min; 7000 rpm), vacuum drying (80°C, 6 h, 0.02 MPa) |
| 2.2.2 | Toluene,  o-xylene,  m-xylene,  p-xylene | 5.0 | 30 | / | 20 kHz (60 W) | 60 | 30 |  |
| 2.2.3 | Toluene,  o-xylene,  m-xylene,  p-xylene | 5.0 | 30 | 5 | / | 60 | 30 |  |
| 2.2.4 | Toluene,  o-xylene,  m-xylene,  p-xylene | 5.0 | 30 | 5 | 20 kHz (60 W) | 60 | 30 |  |

**Table S2.** Numbers of molecules in the molecular-dynamics simulation systems

| System | Solvent | [Bmim][Cl] | SARA |
| --- | --- | --- | --- |
| SARA–toluene–[Bmim][Cl] | 100 | 10 | 20 |
| SARA–m-xylene–[Bmim][Cl] | 100 | 10 | 20 |
| SARA–p-xylene–[Bmim][Cl] | 100 | 10 | 20 |
| SARA–o-xylene–[Bmim][Cl] | 100 | 10 | 20 |

**Table S3.** Calorimetric acoustic power, power density, and energy input at different nominal generator powers

| Nominal generator power (W) | Initial temperature-rise rate (K·s⁻¹) | Actual acoustic power, Pac (W) | Sound energy conversion efficiency (%) | Actual acoustic power density (W·mL⁻¹) | Energy input per unit volume over 20 min (J·mL⁻¹) |
| --- | --- | --- | --- | --- | --- |
| 20 | 0.0431 ± 0.0021 | 6.3 ± 0.3 | 31.5 | 0.180 ± 0.009 | 216.0 ± 10.3 |
| 40 | 0.0875 ± 0.0027 | 12.8 ± 0.4 | 32.0 | 0.366 ± 0.011 | 438.9 ± 13.7 |
| 60 | 0.1326 ± 0.0041 | 19.4 ± 0.6 | 32.3 | 0.554 ± 0.017 | 665.1 ± 20.6 |
| 80 | 0.1757 ± 0.0048 | 25.7 ± 0.7 | 32.1 | 0.734 ± 0.020 | 881.1 ± 24.0 |

Note: The calibration used 35 mL (35 g) of medium and a specific heat capacity of 4.18 J·g⁻¹·K⁻¹. The ultrasound duration was 20 min. Data are reported as mean ± standard deviation (n = 3).

**Derivation of shrinking-core and diffusion-limited extraction models**

To remove differences in fitted plateau recovery among systems from the kinetic comparison, the heavy-oil recovery at time t was converted to the dimensionless extraction fraction shown in Equation (S1):

$X_{t}=\frac{R_{t}}{R_{\infty}}$ (S1)

where Rₜ is the heavy-oil recovery at time t (wt%), R∞ is the fitted plateau recovery (wt%), and Xₜ is the dimensionless extraction fraction ranging from 0 to 1.

For the apparent shrinking-core model, the integral form is given in **Equation (S2)**:

$1-(1-X_{t})^{1/3}=k_{\text{SCM}}t$ (S2)

The corresponding recovery rate expression is given in **Equation (S3):**

$R_{t}=R_{\infty}\left[1-\left(1-k_{\text{SCM}}t\right)^{3}\right]$ (S3)

where k_SCM_ is the apparent shrinking-core-model rate constant (min⁻¹). This comparative model describes the progressive decrease in the extractable oil-film or oil-bearing region during extraction. Because the oil-sand particles are not ideally spherical and the heavy oil is not distributed as a uniform core layer, the fitted model is not interpreted as evidence of a strict geometric shrinking-core mechanism.

A product-layer or internal-diffusion form of the apparent shrinking-core model can also be considered, as shown in Equation (S4):

$1-3(1-X_{t})^{2/3}+2(1-X_{t})=k_{\text{SC},\text{D}}t$ (S4)

where kSC,D is the apparent rate constant for the diffusion-limited shrinking-core form.

**Fick diffusion-controlled extraction model**

Assuming heavy oil components diffuse outward from approximately spherical particles or within oil films, the Fick diffusion model can be expressed as **Equation (S5)**:

$X_{t}=1-\frac{6}{\pi^{2}}\sum_{n=1}^{\infty} \frac{1}{n^{2}}\exp\left(-\frac{n^{2}\pi^{2}D_{\text{eff}}t}{R_{p}^{2}}\right)$ (S5)

For a characteristic diffusion length, the model can be expressed as Equation (S6):

$k_{D}=\frac{D_{\text{eff}}}{R_{p}^{2}}$ (S6)

The model can then be simplified as **Equation (S7)**:

$X_{t}=1-\frac{6}{\pi^{2}}\sum_{n=1}^{\infty} \frac{1}{n^{2}}\exp\left(-n^{2}\pi^{2}k_{D}t\right)$ (S7)

The corresponding recovery rate is given in **Equation (S8):**

$R_{t}=R_{\infty}\left[1-\frac{6}{\pi^{2}}\sum_{n=1}^{\infty} \frac{1}{n^{2}}\exp\left(-n^{2}\pi^{2}k_{D}t\right)\right]$ (S8)

where D_eff_ is the effective diffusion coefficient, R_p_ is the equivalent oil-sand particle radius, and k_D_ is the lumped diffusion parameter (min⁻¹). When accurate particle-size and oil-film-thickness data are unavailable, k_D_ is treated only as a fitted lumped parameter rather than as a directly measured diffusion coefficient.

**Noyes–Whitney liquid-film mass-transfer/dissolution model**

The liquid-film mass-transfer/dissolution process can be represented as **Equation (S9)**:

$\frac{dR_{t}}{dt}=k_{m}\left(R_{\infty}-R_{t}\right)$ (S9)

Integrating Equation (S9) with Rₜ = 0 at t = 0 gives Equation (S10):

$R_{t}=R_{\infty}\left[1-\exp(-k_{m}t)\right]$ (S10)

The dimensionless form is given in **Equation (S11)**:

$X_{t}=1-\exp(-k_{m}t)$ (S11)

In Equation (S11), km is the apparent liquid-film mass-transfer/dissolution rate constant (min⁻¹). This fitted parameter reflects the combined effects of solvent penetration, oil-film swelling, interfacial dissolution, liquid-film diffusion, and transport of dissolved components into the bulk liquid.

First, the experimental recoveries, R_t,exp_, at the different time points were obtained from each kinetic curve, and the late-time plateau was used as the initial estimate of R∞. The apparent shrinking-core, Fickian diffusion, and Noyes–Whitney-type models were then fitted directly to the recovery–time data by nonlinear least squares. Model parameters were determined by minimizing the sum of squared residuals between the experimental and model-predicted values, as shown in Equation (S12):

$\text{SSE=}\sum_{i=1}^{n} \left(R_{i,\text{exp}}-R_{i,\text{cal}}\right)^{2}$ (S12)

Model goodness of fit was evaluated using the coefficient of determination R² (**Equation (S13)**) and RMSE (**Equation (S14)**).

$R^{2}=1-\frac{\sum_{i=1}^{n} \left(R_{i,\text{exp}}-R_{i,\text{cal}}\right)^{2}}{\sum_{i=1}^{n} \left(R_{i,\text{exp}}-\bar{R}_{\text{exp}}\right)^{2}}$ (S13)

$\text{RMSE=}\sqrt{\frac{1}{n}\sum_{i=1}^{n} \left(R_{i,\text{exp}}-R_{i,\text{cal}}\right)^{2}}$ (S14)

Here, R_i,exp_ and R_i,cal_ are the experimental and model-predicted values at the ith time point, respectively, and n is the number of data points. A value of R² closer to 1 and a smaller RMSE indicate better agreement between the model and the experimental data. Model quality was not judged from R² alone; the residual distribution, parameter plausibility, and physical assumptions of each model were also considered.

**Comparative conformational and translational analysis**

The radius of gyration is defined in Equation (S15):

$R_{g}(t)=\left[\frac{\sum_{i=1}^{N} m_{i}\left|\mathbf{r}_{i}(t)-\mathbf{R}_{\text{COM}}(t)\right|^{2}}{\sum_{i=1}^{N} m_{i}}\right]^{1/2}$ (S15)

In Equation (S15), m_i_ and r_i(t)_ are the mass and position of the i atom, respectively, and RCOM is the molecular center of mass. R_g_ describes the spatial compactness of a molecule or cluster. A decrease in R_g_ indicates a more compact configuration, whereas an increase indicates expansion or loosening. Small fluctuations around a stable mean indicate an apparent plateau in conformational compactness over the analyzed time window.

The center-of-mass displacement is defined in Equation (S16):

$\Delta r_{\text{COM}}(t)=\frac{1}{N_{m}}\sum_{m=1}^{N_{m}} \left|\mathbf{R}_{m,\text{COM}}(t)-\mathbf{R}_{m,\text{COM}}(0)\right|$ (S16)

Here, N_m_ is the number of representative molecules of the same type. This parameter describes the average translational displacement of the selected representative molecule within the trajectory. Periodic boundary expansion was applied before the calculation to avoid artificial jumps of molecules crossing cell boundaries. The result value is only used as a trajectory specific descriptor for comparison between models.

The apparent stationary time is defined in operation as the earliest time when the 100 ps moving average of R_g_ remains within ±5% of the mean during the last 200 ps, and the coefficient of variation during the last 200 ps period does not exceed 5%, as shown in equation (S17):

$CV_{R_{g}}=\frac{\sigma_{R_{g},800-1000}}{\bar{R}_{g,800-1000}}\times100\%$ (S17)

Table S4 compares the radius of gyration (R_g_) of the four SARA representative molecules. The values increase with molecular size and structural complexity, from the saturate-like molecule to the asphaltene-like molecule. In each model, the mean Rg during 800–1000 ps was slightly lower than the initial value, suggesting modest local conformational adjustment over the trajectory. These values are descriptive for the finite systems and are not interpreted as equilibrium bulk properties.

The coefficients of variation of R_g_ during the final 200 ps were 1.6–3.0%, showing that R_g_ fluctuated within a relatively narrow range during that window. This apparent stability is limited to the analyzed trajectory and does not demonstrate strict thermodynamic equilibrium. The somewhat larger fluctuations of the resins and asphaltenes indicate less uniform local conformational motion within the same time window.

The fixed-time center-of-mass displacements followed the general order saturates> aromatics>resins>asphaltenes. Across the four solvent models, the saturates value were 18.9–21.8 Å, whereas the asphaltenes value were 6.8–8.1 Å. These numbers describe relative movement within the 1000 ps trajectories and do not represent bulk diffusion. The apparent plateau times are also model-specific descriptors and should not be read as macroscopic relaxation or equilibration times.

Table S4. Comparative trajectory descriptors for SARA representative molecules in the different solvent–[Bmim][Cl] systems

| Solvent system | SARA | Initial R_g_ (Å) | Mean Rg during 800–1000 ps (Å) | Standard deviation of R_g_ (Å) | Change in R_g_ (%) | CV _Rg_ (%) | Mean COM displacement at 1000 ps (Å) | Apparent plateau time (ps) |
| --- | --- | --- | --- | --- | --- | --- | --- | --- |
| Toluene | Saturates | 6.28 | 6.12 | 0.11 | −2.55 | 1.80 | 21.8 | 300 |
| Toluene | Aromatics | 7.82 | 7.55 | 0.15 | −3.45 | 1.99 | 16.4 | 400 |
| Toluene | Resins | 9.74 | 9.38 | 0.22 | −3.70 | 2.35 | 10.8 | 600 |
| Toluene | Asphaltenes | 11.86 | 11.31 | 0.29 | −4.64 | 2.56 | 8.1 | 700 |
| o-xylene | Saturates | 6.31 | 6.18 | 0.12 | −2.06 | 1.94 | 19.6 | 350 |
| o-xylene | Aromatics | 7.88 | 7.63 | 0.16 | −3.17 | 2.10 | 14.8 | 450 |
| o-xylene | Resins | 9.81 | 9.49 | 0.24 | −3.26 | 2.53 | 9.6 | 650 |
| o-xylene | Asphaltenes | 11.93 | 11.42 | 0.31 | −4.27 | 2.71 | 7.2 | 750 |
| m-xylene | Saturates | 6.26 | 6.09 | 0.10 | −2.72 | 1.64 | 20.7 | 300 |
| m-xylene | Aromatics | 7.79 | 7.49 | 0.14 | −3.85 | 1.87 | 15.6 | 400 |
| m-xylene | Resins | 9.69 | 9.31 | 0.21 | −3.92 | 2.26 | 10.2 | 600 |
| m-xylene | Asphaltenes | 11.79 | 11.24 | 0.28 | −4.66 | 2.49 | 7.7 | 700 |
| p-xylene | Saturates | 6.34 | 6.22 | 0.13 | −1.89 | 2.09 | 18.9 | 400 |
| p-xylene | Aromatics | 7.91 | 7.70 | 0.18 | −2.65 | 2.34 | 14.1 | 500 |
| p-xylene | Resins | 9.86 | 9.57 | 0.26 | −2.94 | 2.72 | 8.9 | 700 |
| p-xylene | Asphaltenes | 12.01 | 11.56 | 0.34 | −3.75 | 2.94 | 6.8 | 800 |

**Combined analysis of experiments and molecular dynamics simulations**

The comparison between experiment and MD is made only at the level of qualitative trends. The experimental heavy-oil separation process reflects many coupled effects, whereas the simulations use a finite periodic liquid-phase cell containing a small number of SARA representative molecules, solvents and [Bmim][Cl]. The MD descriptors therefore provide comparative information on relative molecular mobility, local contact, and spatial distribution within the models. They are not used as quantitative predictions of macroscopic recovery, interfacial behavior, or transport rates. The two sets of results are discussed together only to develop a cautious molecular-scale interpretation of the experimental trends.

Table S5 summarizes qualitative links between experimental observations and MD descriptors; it does not imply that each experimental indicator has a directly equivalent simulated quantity. MSD, RDF, concentration profiles, and local configurations are considered only as comparative descriptors. For the toluene system, the statistically significant net interaction estimate (p = 0.023) is identified as a synergistic gain. The p-xylene system showed a borderline statistically significant positive interaction (p = 0.049), whereas the m-xylene and o-xylene systems showed positive but statistically non-significant interaction estimates (p = 0.198 and 0.158, respectively). The simulation results provide interpretive context for the experimental trends, but no numerical correlation or predictive relationship was established. The interfacial experimental indicators were not assigned direct simulation analogues because the periodic liquid-phase MD models contain no mineral surface, aqueous phase, AFM probe, or explicit interface. Consequently, the contact-angle, normalized AFM-force, zeta-potential, and interfacial-tension measurements are interpreted independently from the MD descriptors. The AFM data refer specifically to the hydrophobized SiO₂ model interface, which does not reproduce the mineralogy, morphology, roughness, or wetting heterogeneity of natural oil sands.

The SARA composition results require a joint reading of the MSD and RDF trends. Within the present models, the saturates had a relatively large MSD and weak local correlation with [Bmim][Cl], whereas the resins and asphaltenes have lower MSD values but more evident short-range contact and local concentration fluctuations. This combination is consistent with persistent local association being accompanied by more restricted motion within the simulated time window. MSD describes relative motion over the simulated time window, while RDF and concentration profiles describe local contact and spatial rearrangement. The experimental kinetic parameters and simulation descriptors were considered separately, and no numerical correlation was fitted between them. Together, they provide a trend-level interpretation rather than a quantitative prediction. Accordingly, experimental observations and simulation descriptors are compared only at the qualitative trend level.

Table S5. Qualitative correspondence between experimental indicators and molecular dynamics simulation

| Experimental | Molecular dynamics simulation |
| --- | --- |
| Final extraction rate of heavy oil | Solvent mean square displacement, [Bmim][Cl] mean square displacement, SARA relative migration ability, and concentration distribution |
| Oil-Phase to Solid Ratio (B/S) | SARA concentration distribution, solvent mean square displacement, SARA mean square displacement |
| Solid residual oil content | Solvent mean square displacement, SARA root mean square displacement, and concentration heterogeneity |
| Ultrasound independently extracts increments | Solvent mean square displacement and component concentration redistribution |
| [Bmim] [Cl] Independently extract increments | Components—[Bmim][Cl] radial distribution function, ionic liquid concentration distribution |
| Compound treatment absolutely extracts increments | Mean square displacement of solvents and ionic liquids, radial distribution functions of heavy components |
| Net synergy increments | Solvent migration capacity, local enrichment of ionic liquids, radial distribution functions of heavy components |
| Co-contribution rate | Mean square displacement, radial distribution function, concentration heterogeneity |
| Recovered oil viscosity | Solvent mean square displacement, SARA relative migration ability, and mean square root displacement |
| Recovery oil C/H ratio | Radial distribution functions of resins and asphaltene types, and concentration distribution |
| Saturates content | Saturates mean square displacement, saturates fraction-[Bmim][Cl] radial distribution function |
| Aromatics content | Aromatics mean square displacement, aromatics fraction-[Bmim][Cl] radial distribution function |
| Resins content | Resins mean square displacement, resins fraction-[Bmim][Cl] radial distribution function |
| Asphaltenes content | Asphaltenes mean square displacement, asphaltenes fraction-[Bmim][Cl] radial distribution function |
| Release of resins and asphaltenes | Resins/asphaltenes mean square displacement, resins/asphaltenes radial distribution function of [Bmim][Cl]. |
| Ultrasonic power | solvent migration ability, spatial redistribution of ionic liquids, and degree of configuration adjustment |
| Ultrasound treatment time | Mean square displacement increase, root mean square shift, and concentration distribution evolution |
| Contact angle of residual solid water | [Bmim] [Cl] concentration distribution, local enrichment, and radial distribution functions of components |
| Oil-solid interaction | Radial distribution functions for resins and asphaltenes, local concentration distribution, and configuration rearrangement |
| Absolute value of ζ potential of heavy oil | Radial distribution function of polar components, local concentration distribution of ionic liquids |
| Solid ζ potential | Ionic liquid autocorrelation, [Bmim][Cl] spatial distribution |
| The absolute value of the potential difference between oil and solid phases ζ | Radial distribution function of polar components, fluctuation of ionic liquid concentration |
| Interface tension | [Bmim] [Cl] local enrichment factors, concentration heterogeneity, solvent-ionic liquid distribution |
| Balanced extraction rate R_∞_ | Solvent mean square displacement, SARA relative migration capacity, and concentration redistribution |
| Apparent balance time | Mean square displacement growth rate, fixed-time root mean square displacement |
| Kernel Shrinkage Model Rate Constant k_SCM_ | SARA mean square displacement growth rate and local configuration adjustment |
| Comprehensive Parameters of Filter Diffusion k_D_ | The slope of the mean square displacement of solvents and SARA |
| Liquid film mass transfer–dissolution rate constant k_m_ | Mean square displacement and concentration redistribution of solvents and ionic liquids |
| Noyes–Whitney model determines coefficients | Mean square displacement continuously increases, and component spatial redistribution |
| Empirical first-order dynamic rate constant | Mean square displacement growth rate, fixed-time numerator displacement |
| Empirical first-level dynamic determination coefficient | Energy stability, temperature stability, and continuity of mean square displacement curves |
| Empirical second-order dynamic rate constant | Local migration of SARA and rearrangement of ionic liquid concentration |
| Empirical second-order dynamic determination coefficient | mean square displacement, radial distribution function, and configuration changes |
